# The novel properties of *Kluyveromyces marxianus* glucose sensor/receptor repressor pathway and the construction of glucose repression-released strains

**DOI:** 10.1186/s12934-023-02138-7

**Published:** 2023-07-10

**Authors:** Lingya Wang, Anran Wang, Dongmei Wang, Jiong Hong

**Affiliations:** 1 School of Life Sciences, University of Science and Technology of China, Hefei, Anhui 230027 P. R. China; 2 Hefei National Laboratory for Physical Science at the Microscale, Hefei, Anhui 230026 P. R. China; 3 Biomedical Sciences and Health Laboratory of Anhui Province, University of Science and Technology of China, Hefei, 230027 China

**Keywords:** Glucose repression, SRR pathway, *KmSNF3* disruption, Glucose transporter, Stable KmMTH1 mutant

## Abstract

**Background:**

Glucose repression in yeast leads to the sequential or diauxic utilization of mixed sugars and reduces the co-utilization of glucose and xylose from lignocellulosic biomasses. Study of the glucose sensing pathway helps to construct glucose repression-released yeast strains and enhance the utilization of lignocellulosic biomasses.

**Results:**

Herein, the glucose sensor/receptor repressor (SRR) pathway of *Kluyveromyces marxianus* which mainly consisted of KmSnf3, KmGrr1, KmMth1, and KmRgt1 was studied. The disruption of *KmSNF3* led to a release of glucose repression, enhanced xylose consumption and did not result in deficient glucose utilization. Over-expression of glucose transporter gene restored the mild decrease of glucose utilization ability of *Kmsnf3* strain to a similar level of the wildtype strain but did not restore glucose repression. Therefore, the repression on glucose transporter is parallel to glucose repression to xylose and other alternative carbon utilization. *KmGRR1* disruption also released glucose repression and kept glucose utilization ability, although its xylose utilization ability was very weak with xylose as sole carbon source. The stable mutant of KmMth1-ΔT enabled the release of glucose repression irrespective that the genetic background was *Kmsnf3*, *Kmmth1*, or wildtype. Disruption of *KmSNF1* in the *Kmsnf3* strain or *KmMTH1-ΔT* overexpression in *Kmsnf1* strain kept constitutive glucose repression, indicating that *KmSNF1* was necessary to release the glucose repression in both SRR and Mig1-Hxk2 pathway. Finally, overexpression of *KmMTH1-ΔT* released the glucose repression to xylose utilization in *S. cerevisiae*.

**Conclusion:**

The glucose repression-released *K. marxianus* strains constructed via a modified glucose SRR pathway did not lead to a deficiency in the utilization ability of sugar. The obtained thermotolerant, glucose repression-released, and xylose utilization-enhanced strains are good platforms for the construction of efficient lignocellulosic biomass utilization yeast strains.

**Supplementary Information:**

The online version contains supplementary material available at 10.1186/s12934-023-02138-7.

## Background

Renewable and sustainable lignocellulosic biomasses have increasingly attracted attention as the consumption of fossil fuels not only leads to global warming, but the dwindling fossil resources impact the production of energy and chemicals. It is estimated that as much as 140 billion metric tons of biomass is generated annually from agriculture worldwide. Lignocellulosic biomasses, however, can partly substitute fossil resources, reduce greenhouse gas emissions, and provide renewable energy [1].

The major components of lignocellulosic biomasses are cellulose (35–50%), hemicellulose (20–35%), and lignin (10–25%). The remaining fraction consists of proteins, oils, and ash [2]. The top two fermentable sugars in the cellulolytic enzyme hydrolysates of lignocellulosic biomasses are glucose and xylose, which are derived from cellulose and hemicellulose, respectively. Therefore, the efficient co-utilization of glucose and xylose is necessary to improve the economic benefits of lignocellulosic biomass utilization.

In most xylose assimilation yeasts, glucose repression causes sequential use of glucose and xylose. However, most information regarding glucose repression is obtained from *Saccharomyces cerevisiae*, which has a weak xylose assimilation ability. Therefore, the study of glucose repression to xylose utilization is important in xylose-utilization yeasts, and the glucose repression mechanism may be different from that of *S. cerevisiae.*

**Fig. 1:**
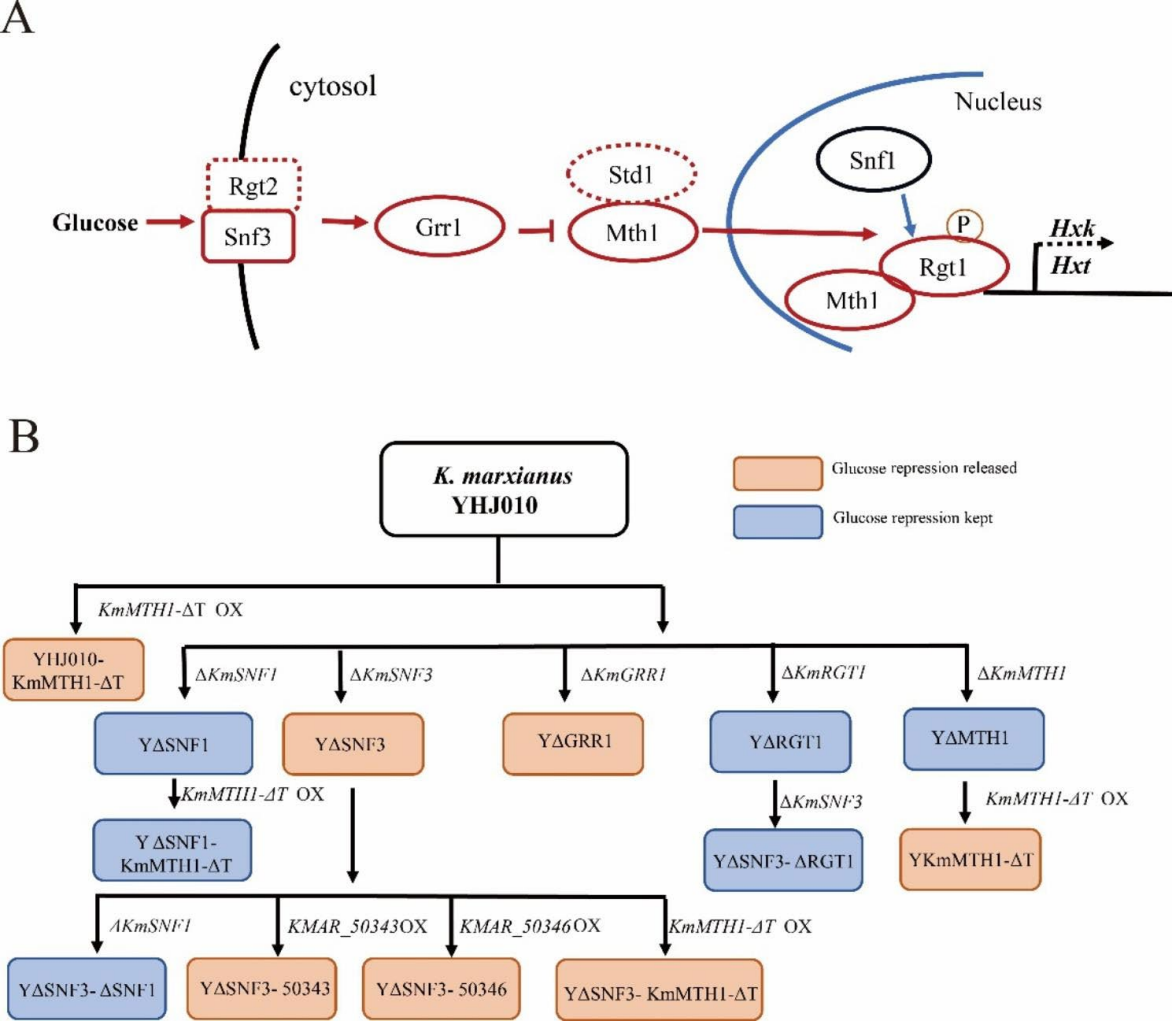
The glucose sensor/receptor repressor (SRR) pathway (**A**) and the main process of K. marxianus strain construction (**B**). Dashed frame: no homologous gene is found in K. marxianus; OX: overexpression; Δ: disruption

In *S. cerevisiae*, the main glucose-sensing and -repression pathways include the sensor/receptor repressor (SRR) pathway (also called the Snf3/Rgt2 pathway), Mig1 -Hxk2 pathway, the cyclic adenosine monophosphate/protein kinase A (cAMP/PKA) pathway [3] and some other regulators. In the Mig1-Hxk2 pathway, after a cell senses a high glucose concentration, Snf1 is deactivated and dephosphorylates Mig1 and Hxk2. Then, Mig1 enters the nucleus and exerts a repression effect on the genes involved in the utilization of alternative carbon sources [3]. In cAMP/PKA pathway, Gpr1 in the cAMP/PKA pathway senses the presence of extracellular glucose and subsequently activates Cyr1, which results in the conversion of adenosine triphosphate into cAMP; then, cAMP activates PKA [4] and the expression of the hexose transporter (Hxt) genes is regulated via the hyperphosphorylation of Rgt1 by PKA [3]. The cAMP-dependent PKA pathway also negatively regulates the activation of Snf1 via phosphorylation [5, 6]. In SRR pathway, Snf3/Rgt2 is proposed to sense the extracellular glucose concentration and transduces a signal via Mth1/Std1 to Rgt1 (Fig. 1A), which regulates the expression of hexose transporters genes thereby regulating the glucose repression [7]. The uptake of glucose in *S. cerevisiae* has been proven to be related to glucose repression, while the mechanism of which remains obscure [8]. Stasyk & Stasyk proposed that the SRR pathway stabilizes the Snf1 complex and protects the functionally important Thr210 of Snf1 from dephosphorylation to fine-tune glucose repression, and that the SRR pathway also affects glucose repression via Mig2 [3].

Disruption or deletion of *SNF3* of *S. cerevisiae* leads to defects in glucose and fructose uptake [9] and the inability of growth on raffinose and sucrose [9]. The *rgt2 snf3* double mutant of *S. cerevisiae* is growth-defective on 2% glucose [10, 11]. The mechanism thereof is that the deletion of *RGT2* and *SNF3* leads to the repression of the *HXT* expression and reduces glucose uptake. Improving glucose uptake in *S. cerevisiae* restores glucose repression [8]. Therefore, it is difficult to construct an efficient-glucose and alternative carbon source co-utilization strain by modifying the SRR pathway in *S. cerevisiae*.

Other than glucose sensing and repression, xylose transport is another limitation in co-utilization of glucose and xylose. The engineered *S. cerevisiae* and other xylose-using yeast species shows a sequential consumption of glucose and xylose due to the transport which has been directly verified by uptake assays for single transporter [12]. Transport affinity of most xylose transporters for glucose is often two orders of magnitude higher than that for xylose, leading to a competitive inhibition by glucose [12]. To co-utilize glucose and xylose, the sugar transport system should also be engineered. The xylose specific and glucose tolerant transporters constructed recently are helpful to resolve this problem [12, 13].

As the xylose assimilation ability of *S. cerevisiae* is weak, most studies on glucose repression do not include the repression to xylose utilization. The xylose utilization of *S. cerevisiae* is improved obviously through genetic engineering, but the informations obtained from the engineered strain currrently does not match the mechanism of glucose repression in xylose-assimilating yeasts. Therefore, special attention needs to be paid to the glucose repression of xylose utilization in such yeasts.

*K. marxianus* is a microorganism “generally regarded as safe” and can assimilate various sugars including glucose, xylose, galactose, and inulin [14]. *K. marxianus* is reported to be the fastest-growing known eukaryotic organism [15] that can lead to high productivity in fermentation. Many *K. marxianus* strains can grow well at temperatures over 45 ^o^C, and maintain a high growth rate at approximately 40 ^o^C [16]. The thermo-tolerance of *K. marxianus* allows for fermentation at elevated temperatures, which is convenient in high-temperature seasons and tropical environments. Additionally in industrial settings, fermentation at elevated temperatures reduces cooling costs, increases fermentation rate, and minimizes the risk of contamination [17, 18].

To efficiently utilize both glucose and xylose from lignocellulosic biomasses, glucose repression should be prevented. Although native *K. marxianus* can utilize xylose, it prefers glucose and causes sequential or diauxic fermentation, resulting in reduced volumetric productivity from sugar mixtures. Several studies on glucose repression in *S. cerevisiae* have been conducted [7, 19–21]; however, the xylose-utilization ability of *S. cerevisiae* without genetic engineering is weak, and glucose effect studies mostly exclude xylose. The mechanism of glucose repression in *S. cerevisiae* is unlikely to apply to *K. marxianus*, particularly the repression of xylose utilization. In our previous study, we found that the glucose repression pathway for xylose is different from that of other sugars, such as galactose [14]. Furthermore, there were fewer genes in the genome of *K. marxianus* (4912 open reading frames for NBRC1777) than those in the genome of *S. cerevisiae*. Therefore, it is unsurprisingly to find evidence of novel glucose-repression mechanisms or properties in *K. marxianus* [14, 22].

In our previous study, we found that in the Mig1-Hxk2 pathway of *K. marxianus*, glucose repression to xylose utilization was possibly not via Mig1 as to other alternative carbon source in the presence of glucose [14]. There might be a novel glucose-sensing mechanism in *K. marxianus*. Herein, the glucose-sensing SRR pathway in *K. marxianus* were analyzed. A single gene-disruption strain (*Kmsnf3*, *Kmgrr1*, *Kmmth1*, or *Kmrgt1*) was constructed to evaluate effect of each gene on glucose repression (Fig. 1B). The glucose repression of strain *Kmsnf3* or *Kmgrr1* to alternative carbon sources utilization was released with only slight decrease glucose consumption ability. Transcriptomic analysis results of the *KmSNF3*-disrupted strain using RNA-seq indicated that in the presence of glucose and xylose, the expression levels of genes related to xylose utilization was upregulated, two main glucose transporter was downregulated and one pentose transporter was upregulated. Overexpression of a stable mutant of KmMth1 (KmMth1-ΔT), from our previous study [23], known for its ability to increased cell growth and elevate pyruvate production, also released the glucose repression (Fig. 1B). The study of crosstalk between the SRR and Mig1-Hxk2 pathways indicated that *KmSNF1* was necessary to release glucose repression in both pathways. Furthermore, the results of overexpressing the glucose-transporter gene in *KmSNF3* disrupted strain showed that improving glucose uptake did not restore the glucose repression (Fig. 1B). Finally, the expression of KmMth1-ΔT in *S. cerevisiae* released the glucose repression to xylose utilization.

## Materials and methods

### Reagents and microorganisms

D-glucose, D-xylose, 2-deoxy-D-glucose (2-DG), and yeast nitrogen base without amino acids (YNB) were purchased from Sangon Biotech Co., Ltd. (Shanghai, China). Yeast extract (LP0021), tryptone (LP0042), and peptone (LP0037) were purchased from Oxoid Ltd. (Basingstoke, UK). Restriction endonuclease and T4 DNA ligase were purchased from Takara Bio Co., Ltd. (Dalian, China). *K. marxianus* YHJ010 is a *TRP1*, *LEU2*, and *URA3* auxotrophic strain derived from NBRC1777 [15]. YPD/YPG medium (10 g/L yeast extract, 20 g/L peptone, 20 g/L glucose, or 20 g/L glycerol) was used for *K. marxianus* culturing. SD medium (6.7 g/L YNB and 20 g/L glucose) with appropriate supplements was used to screen transformants. *Escherichia coli* DH5α was used for gene cloning and was cultivated in lysogeny broth medium (5 g/L yeast extract, 10 g/L tryptone, 10 g/L NaCl). For solid plates, 15 g/L of agar was added to each medium.

### Plasmid construction

The plasmids used herein are shown in the Supplementary Table S1. Plasmids containing gene disruption cassettes were constructed as follows: the DNA fragments, including the up-stream of open reading frame and the downstream of the gene *KmSNF3* (GenBank accession no. MT081965), *KmGRR1* (GenBank accession no. BAP73380), *KmMTH1* (GenBank accession no. BAP71026.1) and *KmRGT1* (GenBank accession no. BAP73047.1) were amplified from the genomic DNA of *K. marxianus* NBRC 1777 with primer pairs KmSNF3F/KmSNF3R, KmGRR1F/KmGRR1R, KmMTH1F/KmMTH1R, and KmRGT1F/KmRGT1R (Supplementary Table S2), respectively. Then the fragments were inserted into pGEM-T easy (Promega, Madison, WI, USA). The resultant plasmids were named pKmSNF3, pKmGRR1, pKmMTH1, and pKmRGT1 (Supplementary Table S1), respectively. The frames and parts of the genes were then amplified with primer pairs: dKmSNF3F/dKmSNF3R, dKmGRR1F/dKmGRR1R, dKmMTH1F/dKmMTH1R, and dKmRGT1F/dKmRGT1R (Supplementary Table S2) to remove parts of the corresponding ORF and linearization. The expression cassette of ScURA3 was amplified from the plasmid YEUGAP using the primer pair: SCURA3-SMAI-FULL-F/SCURA3-SMAI-FULL-R (Supplementary Table S2), and digested with *Sm*aI [14]. Then, the *ScURA3* expression cassette was ligated with the aforementioned linearized plasmids. The resultant plasmids were pKmSNF3-U, pKmGRR1-U pKmMTH1-U, and pKmRGT1-U (Supplementary Table S1), each of which contained corresponding disruption cassettes.

The plasmid for the expression of the putative sugar transporter gene was constructed as follows: The ORF DNA fragment of *KMAR_10531*, *KMAR_50343*, or *KMAR_50346*, which encode the putative sugar transporter, was amplified from the genomic DNA of *K. marxianus* NBRC 1777 with the primer pair Km10531-EcoRI-F/Km10531-NotI-R, Km50343-EcoRI-F/Km50343-NotI-R or Km50346-EcoRI-F/Km50346-NotI-R (Supplementary Table S2), respectively. The fragments were then digested with *Eco*R I and *Not* I and inserted into the same restriction enzyme digested YEGAP [24]. The obtained plasmids were named YEGAP-10,531, YEGAP-50,343, and YEGAP-50,346, respectively (Supplementary Table S1).

### Strains construction

The strains used herein are listed in Table 1, and the strain construction procedure is shown in Fig. 1.

The strains in which a single gene of the SRR pathway was disrupted (Table 1; Fig. 1) were constructed to analyze their effect on glucose repression. The disruption cassettes were amplified from the plasmids pKmSNF3-U, pKmGRR1-U, pKmRGT1-U, and pKmMTH1-U with the primer pairs KmSNF3F/KmSNF3R, KmGRR1F/KmGRR1R, KmRGT1F/KmRGT1R, and KmMTH1F/KmMTH1R (Supplementary Table S2), and transformed into *K. marxianus* YHJ010 to disrupt target genes through homologous recombination by LiOAc transformation [24, 25]. The transformants were selected on an SD medium plate containing leucine and tryptophan and the disruption was confirmed by colony-PCR using the same primers as described above. The strains from which only one longer DNA fragment was amplified were positive. The obtained strains were named YΔSNF3, YΔGRR1, YΔRGT1, and YΔMTH1 (Table 1; Fig. 1).

**Table 1:** Yeast strains used in this study

Strains	Description	References
***S. cerevisiae***		
EBY.VW4000	*MATαleu2-3,112 ura3-52 trp1-289 his3-Δ1 Mal2-8c SUC2 hxt17Δhxt13Δ::loxPhxt15Δ::loxPhxt16Δ::loxPhxt14Δ::loxPhxt12Δ::loxPhxt9Δ::loxPhxt11Δ::loxPhxt10Δ::loxPhxt8Δ::loxPhxt514::loxPhxt2Δ::loxPhxt367Δ::loxPgal2Δstl1Δ::loxPagt1Δ::loxPydl247wΔ::loxPyjr160cΔ::loxP*	[26]
W303-1 A	*MAT***a***leu2-3*,*112 ura3-1 trp1-1 his3-11*,*15 ade2-1 can1-100 mal10 GAL SUC2*	ATCC 208,352
YW303-1 A-*KmMTH1-ΔT*)	*S. cerevisiae* W303-1 A, *KmMTH1-ΔT*	This study
YEBYVW4000- 10,531	EBY.VW4000, *KMAR_10531*	This study
YEBYVW4000-50343	EBY.VW4000, *KMAR_50343*	This study
YEBYVW4000-50346	EBY.VW4000, *KMAR_50346*	This study
***K. marxianus***		
NBRC1777	*K. marxianus* wildtype	[24]
YHJ010	NBRC1777, Δ*KmURA3*::*KAN*^*R*^, Δ*KmLEU2*::*HISG*, Δ*KmTRP1*::*HISG*	[24]
YWD005	YHJ010, *ScURA3*	[27]
YWD010	YHJ010, *ScURA3*, *ScTRP1*	[27]
YΔRGT1	YHJ010, *Kmrgt1*::*ScURA3*	[14]
YΔSNF1	YHJ010, *Kmsnf1*::*ScURA3*	[14]
YΔSNF3	YHJ010, *Kmsnf3*::*ScURA3*	This study
YΔSNF3-U	YΔSNF3, *ura3*	This study
YΔSNF3-ΔSNF1	YHJ010, *Kmsnf3*, *Kmsnf1*::*ScURA3*	This study
YΔMTH1	YHJ010, *Kmmth1*::*ScURA3*	This study
YΔMTH1-U	YΔMTH1, *ura3*	This study
YΔGRR1	YHJ010, *Kmgrr1*::*ScURA3*	This study
YΔRGT1-U	YΔRGT1, *ura3*	This study
YΔSNF3-ΔRGT1	YHJ010, *Kmrgt1, Kmsnf3*::*ScURA3*	This study
YΔSNF3- KmMTH1-ΔT	YHJ010, *Kmsnf3, KmMTH1-ΔT*	This study
YKmMTH1-ΔT	YHJ010, *Kmmth1, KmMTH1-ΔT*	This study
YHJ010-KmMTH1-ΔT	YHJ010, *KmMTH1-ΔT*	This study
YΔSNF3 -50,343	YHJ010, *Kmsnf3, KMAR_50343, ScTRP1*	This study
YΔSNF3 -50,346	YHJ010, *Kmsnf3, KMAR_50346, ScTRP1*	This study
YΔSNF3-TRP1	YHJ010, *Kmsnf3, ScTRP1*	This study
YΔSNF1-U	YΔSNF1, *ura3*	This study
YΔSNF1-KmMTH1-ΔT	YΔSNF1, *KmMTH1-ΔT*	This study

*KmSNF1* in YΔSNF3 was disrupted to construct a double-disrupted strain. This strain was used to evaluate the crosstalk between the SRR and Mig1-Hxk2 pathways. The *ScURA3* in *K. marxianus* YΔSNF3 was disrupted to recover the URA3 selection marker using the *ScURA3* disruption cassette, which was amplified from pMD18T-ΔScURA3 as described previously [28]. After *K. marxianus* YΔSNF3 was transformed with the *ScURA3* disruption cassette, the transformants were screened on SD medium containing 5-fluoroorotic acid (5-FOA). The strain that was obtained was YΔSNF3-U. Then, the *KmSNF1*disruption cassette was amplified from pKmSNF1-U [14] with primers KmSNF1F and KmSNF1R (Supplementary Table S2) and transformed into YΔSNF3-U to disrupt *KmSNF1*. After screening on an SD medium containing leucine and tryptophan, and colony-PCR confirmation, the *Kmsnf*, *Kmsnf3* strain YΔSNF1-ΔSNF3 was obtained (Table 1).

The *Kmsnf3*, *Kmrgt1* strain was constructed to evaluate the effect of KmRgt1 on the SNF3 pathway. *ScURA3* in *K. marxianus* YΔRGT1 was disrupted as described above. The obtained strain was YΔRGT1-U. The *KmSNF3* disruption cassette was then amplified from pKmSNF3-U [14] using primers KmSNF3F and KmSNF3R (Supplementary Table S2) and transformed into YΔRGT1-U to disrupt *KmSNF3*. The *Kmrgt1*, *Kmsnf3* strain, YΔSNF3-ΔRGT1, was obtained (Table 1).

Stable Mth1 mutant (Mth1-ΔT) of *S. cerevisiae* has an internal deletion in the open reading frame. The deleted region encodes putative proline (P), glutamic acid (E), serine (S) and threonine (T) enriched (PEST) sequences and a phosphorylation site for YCK1 [29]. In our previous study, a similar mutant KmMth1-ΔT was proven to enhance the glucose utilization in a *KmPDC1*-disrupted *K. marxianus* strain [23]. Here, several *KmMTH1-ΔT* overexpression strains were constructed to evaluate whether this stable mutant could maintain or lead to the release of glucose repression. The *ScURA3* in YΔSNF3 or YΔMTH1 was disrupted with the *URA3* disruption cassette [28], and strains YΔSNF3-U or YΔMTH1-U were obtained in which the selection marker was recovered. Then, YΔSNF3-U, YΔMTH1-U or YHJ010 were transformed with the plasmid pZB013 [23], which contained the *KmMTH1-ΔT* expression cassette. The obtained strain was YΔSNF3-KmMTH1-ΔT, YKmMTH1-ΔT or YHJ010-KmMTH1-ΔT.

*KmMTH1-ΔT* was also expressed in YΔSNF1 strain to evaluate the effect of the stable KmMTH1 mutant on glucose repression in *KmSNF1*-disrupted strain. The *ScURA3* in YΔSNF1 [14] was disrupted to recover the Ura3 selection marker using the *ScURA3* disruption cassette as described previously [28]. The obtained strain was YΔSNF1-ΔURA3. Thereafter, plasmid pZB013 was transformed into YΔSNF1-ΔURA3 and strain YΔSNF1-KmMTH1-ΔT was obtained.

The strains expressing the putative sugar transporter genes, *KMAR_50343* or *KMAR_50346*, were constructed using *K. marxianus* YΔSNF3 (Fig. 1A). These strains were used to determine the effects of glucose transport on glucose repression. Plasmids YEGAP-50,343 or YEGAP-50,346 were transformed into *K. marxianus* YΔSNF3 and screened on an SD medium supplemented with leucine. The obtained strains were named YΔSNF3-50343 and YΔSNF3-50346, respectively (Table 1; Fig. 1A).

The putative sugar transporter genes, *KMAR_50343*, *KMAR_50346* or *KMAR_10531*, were expressed in *S. cerevisiae* to evaluate their function. These genes were selected due to their high expression level and markedly changed expression following *KmSNF3* disruption. Plasmids YEGAP-10,531, YEGAP-50,343 or YEGAP-50,346 were transformed into *S. cerevisiae* EBY.VW4000, in which all HXTs were deleted (gift from Professor Eckhard Boles) [26], and YEBYVW4000-50343, YEBYVW4000-50346, and YEBYVW4000-10531 were obtained (Table 1; Fig. 1B).

A *KmMTH1-ΔT*-expressing *S. cerevisiae* was constructed to evaluate its effect on glucose repression. Plasmid pZB013 was transformed into *S. cerevisiae* W303-1 A and screened on an SD medium supplemented with leucine, tryptophan, histidine, and adenine, and the strain YW303-1 A-KmMTH1-ΔT was obtained (Table 1; Fig. 1B).

### Evaluation of glucose repression of constructed strains on a 2-DG plate

As glucose analog 2-DG can be taken up by yeast cells and cause glucose repression [30], the glucose repression of the constructed strains was determined by dropping the cells on the medium containing 2-DG and various carbon sources. All strains were precultured in a YPD medium. After overnight cultivation at 37 °C and with 250 rpm shaking, the cells were collected by centrifugation at 5000×g for 5 min and resuspended in sterilized water. The diluted cell suspension was spotted on YP plates containing various carbon sources (glucose, xylose, lactose, sucrose, raffinose, or glycerol), with or without 0.01% 2-DG. Then, the plates were incubated at 37 °C for *K. marxianus* and 30 °C for *S. cerevisiae*.

### Evaluating the growth of the *Kmsnf3* strain on low- or high-concentration glucose plates

As Snf3 is a glucose sensor of low-concentration glucose, *Scsnf3* strain of *S. cerevisiae* can lead to a deficient growth in a low concentration of glucose [31]. The growth of YΔSNF3 in the presence of low or high-concentration glucose was determined to evaluate the function of KmSnf3. *K. marxianus* YWD005 (YHJ010 + *ScURA3*, control with the same auxotrophic type) or YΔSNF3 was streaked on an SD plate medium containing 0.1% or 2% glucose, and the plates were cultivated at 37 ^o^C for 2 days.

### Evaluating the glucose and xylose consumption of the constructed strains

As our study focused on glucose repression to xylose utilization, the glucose and xylose consumption of the constructed strains during culturing was analyzed. YWD005 (control), YΔSNF3, YΔGRR1, YΔMTH1, and YΔRGT1 cells were pre-cultivated in 5 mL of YPD medium at 42 °C overnight. Because elevated temperatures will be used in futural application, taking advantage of the thermotolerant nature of *K. marxianus*, 42 °C was used for the cultivation in liquid medium. Then, the cells from pre-cultivation were transferred into 250-mL Erlenmeyer flasks containing 50 mL of YPD (2% glucose), YPX (2% xylose), and YPDX (2% glucose + 2% xylose) media with an initial OD_600_ of 0.5 and cultivated at 42 °C with shaking at 250 rpm in an orbital shaker. Growth with 2% xylose, 2% glucose, and 2% xylose + 2% glucose was determined by monitoring the OD_600_. The sugar consumption during the growth was monitored by high-performance liquid chromatography (HPLC).

YΔSNF3-ScTRP1, YΔSNF3-50343, YΔSNF3-50346, and YWD010 (YHJ010 + ScURA3 + ScTRP1, control with same auxotrophic type) were pre-cultivated in 5 mL of YPD medium at 42 °C overnight. Then, the cells were transferred into 250-mL Erlenmeyer flasks containing 50 mL of YPDX (2%glucose + 2% xylose, pH6.0) medium with an initial OD_600_ of 0.5 and cultivated at 42 °C with shaking at 250 rpm in an orbital shaker. Growth was determined by measuring the OD_600_. The sugar consumption during growth was monitored by HPLC.

### Transcriptome analysis

RNA-seq was used to analyze the changes in gene expression before and after *KmSNF3* disruption in the presence of glucose and xylose. *K. marxianus* YΔSNF3 and YWD005 were pre-cultivated in 5 mL of YPD medium at 42 °C overnight. Then, the cells were transferred into 250-mL Erlenmeyer flasks containing 30 mL of YPDX medium (2% glucose and 2% xylose) with an initial OD_600_ of 1.0 and cultivated at 42 °C with shaking at 250 rpm in an orbital shaker until the OD_600_ = 6.0 before the glucose was exhausted. Yeast cells were then recovered and stored at -80 °C for RNA isolation.

The total RNA of each sample was extracted and cDNA libraries were prepared as previously described [22]. The resulting cDNA library products were then shotgun sequenced (101 bp paired-end read) using the Illumina HiSeq 4000 instrument (Illumina, San Diego, CA, USA) through a customer sequencing service (Majorbio Co., Ltd, Shanghai, China). Annotation and bioinformatics analyses were performed as previously described [22]. For differential gene expression analysis, transcripts per kilobase of the exon model per million mapped reads (tpm) were used as a value of normalized gene expression. Genes were considered differentially expressed in a given library when a *p*-value < 0.05 and a greater than two-fold change in expression across libraries was observed.

### Real-time PCR

Quantitative real-time PCR (qRT-PCR) was used to evaluate the expression of the key genes for alternative carbon source utilization in the presence of glucose. After *K. marxianus* strains were overnight cultivated in YPD medium at 42 °C and with 250 rpm shaking, the cells were inoculated into YP medium containing 20 g/L glucose, 20 g/L glucose and 20 g/L alternative carbon source, 20 g/L alternative carbon source until the OD_600_ reached 6.0. The alternative carbon sources used were lactose, sucrose, xylose, raffinose or glycerol. The cells were collected by centrifugation at 5000×g for 5 min and total RNA was extracted using the Yeast Total RNA Isolation Kit (Sangon Biotech, Shanghai). The genomic DNA in the sample was removed by 4×DN Master Mix with gDNA Remover. The cDNA was reverse-transcribed using the ReverTra Ace® qPCR RT Master Mix with gDNA Remover (Toyobo, Japan). The expression levels of *KmXYL1*, *KmXYL2*, *KmXYL3*, *KmLAC4*, *KmINU1*, *KmGUT1*, *KmGUT2*, and *KmHXK1* (hexokinase 1) were analyzed in the presence of glucose, alternative carbon sources or a glucose and alternative carbon source mixture. The primers used for RT-PCR are listed in Supplementary Table S2.

## Evaluation of the function of sugar transporter gene expressed *S. cerevisiae* strains

*S. cerevisiae* EBY.VW4000, YEBYVW4000-50343, YEBYVW4000-50345, and YEBYVW4000-10531 (Table 1) were streaked on an SD medium containing 20 g/L maltose (SM) or 20 g/L glucose (SD). The plates were incubated at 30 °C for 2 days.

### Statistical analysis

All experiments were run three times, and the standard error of the mean was represented in the figures as error bars.

## Results and discussion

### Disruption of *KmSNF3* in *K. marxianus* led to a release of glucose repression and maintained normal glucose and alternative carbon source utilization ability

After the single gene disruption strains of the SRR pathway were obtained (Fig. 1; Table 1), cells of these strains were dropped on yeast peptone (YP) medium plates containing various carbon sources, with or without the presence of 2-DG. 2-DG is a stable glucose analogue that is taken up into cells by hexose transporters and phosphorylated but cannot be fully metabolized, therefore, it is a convenient reagent for screening of mutants defective in glucose repression [30].

**Fig. 2:**
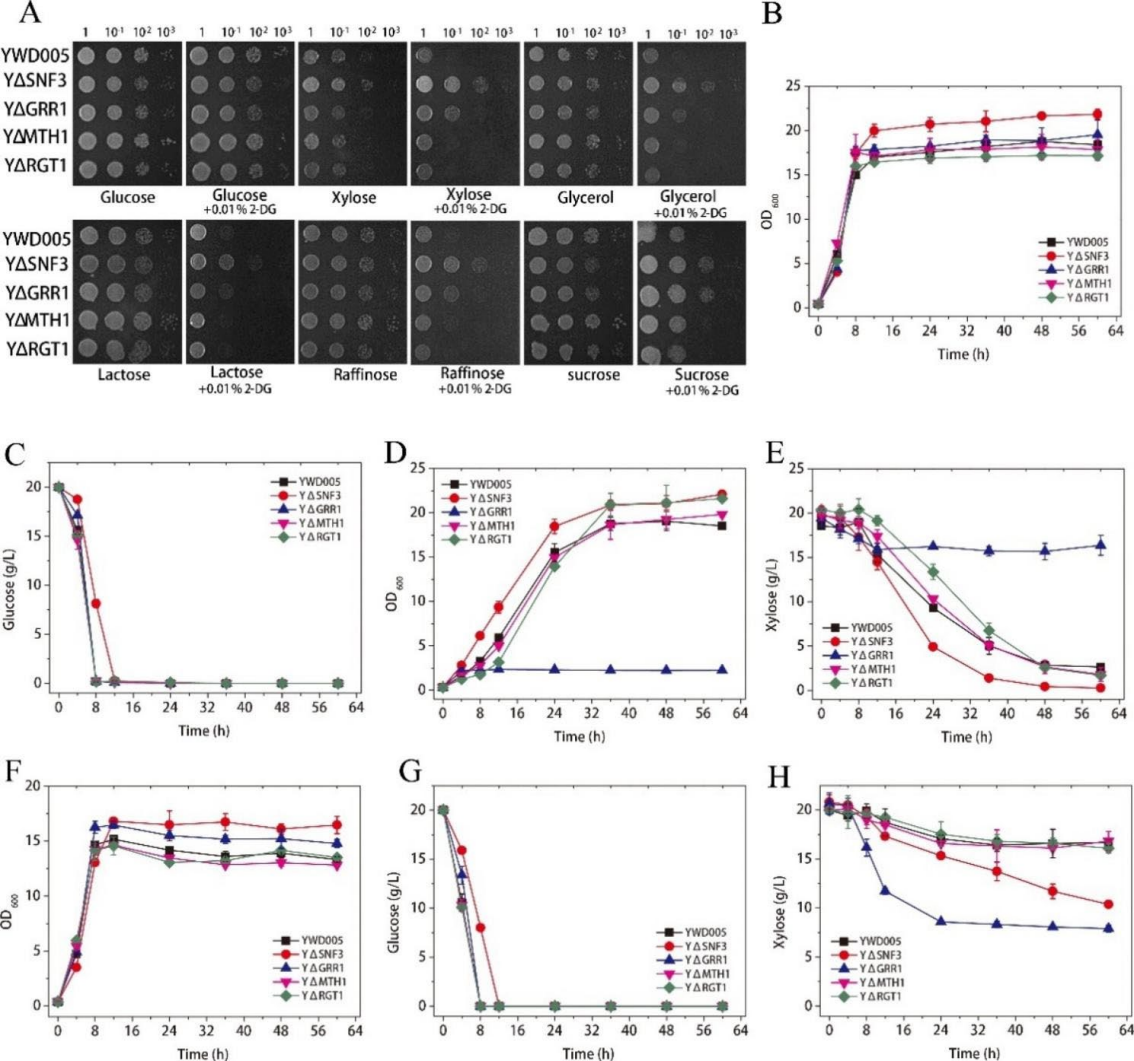
The evaluation of glucose repression in YWD005, YΔSNF3, YΔGRR1, YΔMTH1, and YΔRGT1 strains. The release of glucose repression in the presence of 2-DG (**A**); the growth and sugar consumption of the culture at 42 ^o^C with glucose (**B**, **C**), xylose (**D**, **E**), and a glucose-xylose mixture (**F**, **G**, and **H**). YWD005 served as a non-disrupted control. The error bars represent the standard deviations calculated from triplicate experiments

The growth of YΔSNF3 was similar to that of the non-disrupted control (YWD005, Table 1) on the YPD solid medium (Fig. 2A). Furthermore, YΔSNF3 grew similarly to YWD005 on alternative carbon sources (xylose, glycerol, lactose, raffinose, or sucrose) and grew even better than YWD005 on alternative carbon sources in the presence of 2-DG (Fig. 2A). Therefore, *KmSNF3* disruption released the glucose repression without observable changes in the growth on the glucose or alternative carbon sources. These results were unexpected. In *S. cerevisiae*, the *snf3* mutant caused defects in the uptake of glucose and fructose and was unable to grow in raffinose and sucrose [9]. The *rgt2 snf3* double mutant of *S. cerevisiae* was growth defective in 2% glucose [10, 11]. As only the *SNF3* homolog was found in the genome of *K. marxianus*, *Kmsnf3* disruption in *K. marxianus* was thought to correspond to *rgt2 snf3* double disruption in *S. cerevisiae.* Unexpectedly, the *Kmsnf3* resulted in the derepression of glucose repression and did not lead to growth defects in the presence of glucose, sucrose, and raffinose (Fig. 2A).

### *KmSNF3* disruption led to improved growth in the presence of glucose and enhanced xylose utilization in liquid media

The growth of YΔSNF3 and YWD005 were further evaluated in a liquid medium with glucose, xylose, or a glucose-xylose mixture. The *Kmsnf3* strain (YΔSNF3) grew better than the non-disrupted control (YWD005) and accumulated more biomass in the liquid medium with glucose, with a final OD_600_ of 21.83 and 18.74, respectively (Fig. 2B). The glucose consumption rate of YΔSNF3 and YWD005 was 1.67 g/(L·h) and 2.50 g/(L·h), respectively (Fig. 2C). Although the glucose consumption of YΔSNF3 was slower than that of YWD005, YΔSNF3 still rapidly consumed glucose.

Furthermore, YΔSNF3 grew on xylose at an initial growth rate of 0.28 /h, and the final OD_600_ reached 20.88 (Fig. 2D) in 36 h, whereas YWD005 grew on xylose at an initial growth rate of 0.25 /h, and the final OD_600_ reached 18.77 (Fig. 2D). The xylose-utilization ability of YΔSNF3 was higher than that of YWD005 (Fig. 2E). YΔSNF3 consumed 18.26 g/L xylose at a xylose-consumption rate of 0.51 g/(L·h) and consumed almost all the xylose in 48 h, whereas, YWD005 only consumed 13.55 g/L of xylose at a xylose-consumption rate of 0.38 g/(L·h) (Fig. 2E). These results suggest that the disruption of *KmSNF3* improved xylose utilization by *K. marxianus*.

Finally, when the glucose-xylose mixture was used as a carbon source, the OD_600_ of YΔSNF3 reached 16.81, whereas the OD_600_ of YWD005 was 15.17 at 12 h (Fig. 2F). The glucose consumption rates of YΔSNF3 and YWD005 in the glucose-xylose mixture were similar to those in the presence of glucose alone (Fig. 2C and G). In the presence of glucose, YΔSNF3 consumed more xylose in the mixed sugar in 60 h (10.44 g/L) compared to 3.31 g/L consumed by YWD005. These results indicated that the disruption of *KmSNF3* enhanced xylose utilization by *K. marxianus* in the presence of glucose (Fig. 2H). Before the glucose was completely consumed (in 12 h) (Fig. 2H), the slow xylose consumption is due to competitive inhibition of glucose to xylose transport. After 12 h, xylose was used by the readily expressed xylose metabolism enzymes in YΔSNF3 and showed an enhanced xylose utilization.

In *S. cerevisiae*, Snf3 and Rgt2 are glucose sensors that respond to low and high concentrations of glucose, respectively. The disruption of *SNF3* in *S. cerevisiae* leads to a deficiency in growth in low-concentration glucose [31]. To determine whether KmSnf3 responded to low-concentration glucose, the glucose utilization ability of YΔSNF3 was evaluated on low (0.1%) and high glucose (2%) synthetic media. The results showed that YΔSNF3 grew similarly to the control strain (YWD005) (Supplementary Fig. S1). Therefore, the disruption of *Kmsnf3* did not lead to a deficient utilization ability of low-concentration glucose (Supplementary Fig. S1).

In other reports, the glucose utilization ability of glucose repression-released *K. marxianus* strains is markedly decreased [14, 32]. The glucose consumption rate of *K. marxianus* SBK-1, which is a catabolite repression-alleviated strain, decreases significantly compared to that of the parental strain (from 5.66 g/(L·h) to 0.34 g/(L·h)) [32]. In our previous study, the *Kmhxk1* strain releases glucose repression with a significantly decreased glucose-assimilating ability [14]. Expressing *KmGLK* improves glucose assimilation ability and maintains the release of glucose repression, but the strain loses fructose utilization ability [14, 27]. Therefore, the glucose repression-released YΔSNF3, which kept the glucose utilization ability and improved xylose utilization ability, could be useful for lignocellulosic biomass utilization.

The ethanol accumulations of YΔSNF3 and YWD005 during the cultivation were also determined. YWD005 produced 6.82 g/L ethanol at 8th h with glucose, whereas, YΔSNF3 produced less ethanol (6.14 g/L) with longer time (12 h) (Fig. S2). In the case of xylose as carbon source, only very small amount of ethanol accumulated (YWD005: 0.47 g/L at 36th h, YΔSNF3: 0.40 g/L at 60th h) (Fig. S2). When glucose and xylose mixture was used as carbon source, Y ΔSNF3 produced 5.63 g/L ethanol at 12th h and YWD005 produced 5.54 g/L ethanol at 8th h. The ethanol accumulation of Y ΔSNF3 was slower than that of YWD005, and was consistent with the slower sugar consumption of Y ΔSNF3 than YWD005.

### Transcriptome analysis indicated that glucose repression in YΔSNF3 was released

Although a spot assay in the presence of 2-DG is a traditional method for glucose-repression determination, the changes in gene expression still need to be verified. YΔSNF3 and YWD005 were cultivated with mixture of glucose and xylose, RNA-seq was used to analyze the transcriptional changes of KmSNF3-disruption in response to the glucose repression with YWD005 as control strain. In GO enrichment analysis, among those significantly enriched pathways, single-organism process, small molecule metabolic process, single-organism biosynthetic process, oxoacid metabolic process, carboxylic acid metabolic process, and organic acid metabolic process were among the top 40 GO terms with significant enrichment (Supplementary Fig. S3A).

As to KEGG enrichment analysis, among those significantly enriched pathways, citrate cycle (TCA cycle), pyruvate metabolism, glycolysis/gluconeogenesis, and many amino acids metabolism and biosynthesis such as alanine, aspartate, glutamate, phenylalanine, tyrosine, tryptophan, and arginine etc., were in the top 40 pathways with significant enrichment. Galactose metabolism, fructose and mannose metabolism were also in this top 40 pathways (Supplementary Fig. S3B). These results suggest that these common DEGs have relationships with glucose repression.

As our study focused on glucose-xylose co-utilization, and *K. marxianus’* ability to utilize xylose, the transcriptome analysis was mainly related to glucose repression, particularly xylose utilization. The expressions of xylose utilization genes, including xylose reductase (*KmXYL1*), xylitol dehydrogenase (*KmXYL2*), and xylulokinase (*KmXYL3*), improved by Log_2_FC values of 3.71, 4.14, 1.94, respectively (Supplementary Table S3).

As xylose metabolism occurs via the pentose phosphate pathway (PPP), which is downstream of xylose catabolism, the expression of genes involved in PPP was also analyzed. There was no obvious change, except for the upregulated expression of glucose-6-phosphate 1-dehydrogenase (*ZWF*) (Log_2_FC: 1.30; Supplementary Table S3). The disruption of *KmSNF3* did not lead to enhanced PPP activity.

The expression of genes encoding the enzymes involved in alternative carbon source utilization was also analyzed. The expressions of the β-glucosidase (*KmBGL1*) and inulinase (*KmINU1*) genes that correspond to cellobiose or inulin (also sucrose, and raffinose) utilization, respectively, did not change significantly. This was possibly due to the absence of an inducer (carbon source) in the medium.

The expression of genes related to lactose utilization, such as galactokinase (*KmGALK*), lactose regulatory protein LAC9 (*KmGAL40*), and galactose/lactose metabolism regulatory protein (*KmGAL80*), was upregulated (Log_2_FC: 1.95, 3.35, and 2.21, respectively); however, the expression of β- galactosidase (*KmLAC4*) was not changed.

In *K. marxianus*, four alcohol dehydrogenase genes (*ADH*) were found in the genome. KmAdh1 and KmAdh2 are cytosolic enzymes, whereas KmAdh3 and KmAdh4 are mitochondrial ADHs [33]. In the presence of glucose and xylose, compared with the control strain (YWD005), *KmADH1* and *KmADH3* were upregulated (Log_2_FC 6.10 and 2.99) and *KmADH2* was downregulated (Log_2_FC -3.82), whereas there was no significant difference of *KmADH4* in YΔSNF3. This is possible because KmADH3 and KmADH4 cannot catalyze the conversion of acetaldehyde to ethanol, and they are the enzymes involved in ethanol consumption. Although the *Kcat*/*K*m of KmAdh1 (4.3 × 10^3^ min^− 1^ mM^− 1^) and KmAdh2 (2.7 × 10^3^ min^− 1^ mM^− 1^) with acetaldehyde as the substrate is relatively high, KmAdh1 has a low *Kcat*/*K*m (5.2 min^− 1^ mM^− 1^) with ethanol as the substrate, in contrast to KmAdh2 (2.1 × 10^2^ min^− 1^ mM^− 1^). Therefore, KmAdh2 is a respondent to ethanol production [33]. The decreased expression of KmAdh2 (Log_2_FC: -3.82) and increased expressions of KmAdh1 (Log_2_FC: 6.10) and Adh3 (Log_2_FC: 2.99) indicated that the expression of genes related to ethanol production decreased and those related to ethanol utilization increased in YΔSNF3.

Mig1-Hxk2, SRR, and cAMP/PKA pathways are all glucose-sensing. In the Mig1-Hxk2 pathway, the expressions of *KmHXK1* and *KmMIG1* in YΔSNF3 was downregulated with a Log_2_FC value of -2.20 or -3.90, respectively, compared to those in YWD005 (Supplementary Table S3). In our previous results, the disruption of *KmHXK1* or *KmMIG1* led to the release of glucose repression, although the repression of xylose utilization was not released via *KmMIG1* disruption [14]. These results were consistent with the phenotype in which the repression to alternative carbon source utilization was alleviated. There was no obvious change in the expression of the *KmSNF1* complex (Supplementary Table S3). The activity of KmSNF1 may be regulated through phosphorylation, whereas the expression level is not important. In the cAMP-PKA pathway, the expression of most genes was not regulated, except for those of *KmGPA2* and *KmTPK2* (Log_2_FC: 2.17 and 1.27, respectively). In the SRR pathway, the expression of *KmRGT1* were upregulated (Log_2_FC: 1.58), while *KmMTH1* was downregulated (Log_2_FC: -1.59) in YΔSNF3 (Supplementary Table S3). However, the activity and degradation of KmRgt1 and KmMth1 were dependent on protein modification, therefore, the change in the expression level was less important for glucose repression.

The expression levels of possible sugar transporters were subsequently analyzed. As shown in Supplementary Table S3, 14 sugar transporter genes were significantly regulated. The expression levels of seven high-affinity glucose transporters, two lactose permeases, one low-affinity glucose transporter, and one sugar transporter, STL1, were upregulated, while two *KmHXT2* and one low-affinity glucose transporter were downregulated (Supplementary Table S3). Among these transporter genes, *KMAR_50343*, *KMAR_50346*, and *KMAR_10531* had abundant transcripts (high transcripts per kilobase of the exon model per million mapped reads (tpm)) and changed significantly (Supplementary Table S3). The expression of *KMAR_50343* decreased from 1606.97 to 135.28 tpm with a Log_2_ FC value of -3.48 and the expression of *KMAR_50346* decreased from 1325.97 to 2.87 tpm with a Log_2_ FC value of -8.27, whereas the expression of *KMAR_10531* increased from 10.04 to 2729.2 tpm with a Log_2_ FC value of 8.22. The expression levels of other transporter genes were relatively low, even with significant regulation. In *S. cerevisiae*, glucose is sensed by SNF3/RGT2 and the signal is transmitted to Rgt1 via Mth1/Std1, then Rgt1 is released from the promoters of *HXT* genes, and *HXT* is expressed. However, in the absence of glucose in the medium, the expression of *HXT* genes is repressed [34]. The downregulation of *KMAR_50343* and *KMAR_50346* in YΔSNF3 reflects a no-glucose status. Upregulated *KMAR_10531* encodes a pentose transporter [35] that is possibly induced by xylose.

### Results of real time-PCR confirmed that *KmSNF3* disruption released glucose repression

Although the RNA-seq results verified that the disruption of *KmSNF3* resulted in the release of glucose repression to xylose utilization, the release of glucose repression to xylose and other alternative carbon sources utilization were further verified by determining the expression level of related genes by RT-PCR.

**Fig. 3:**
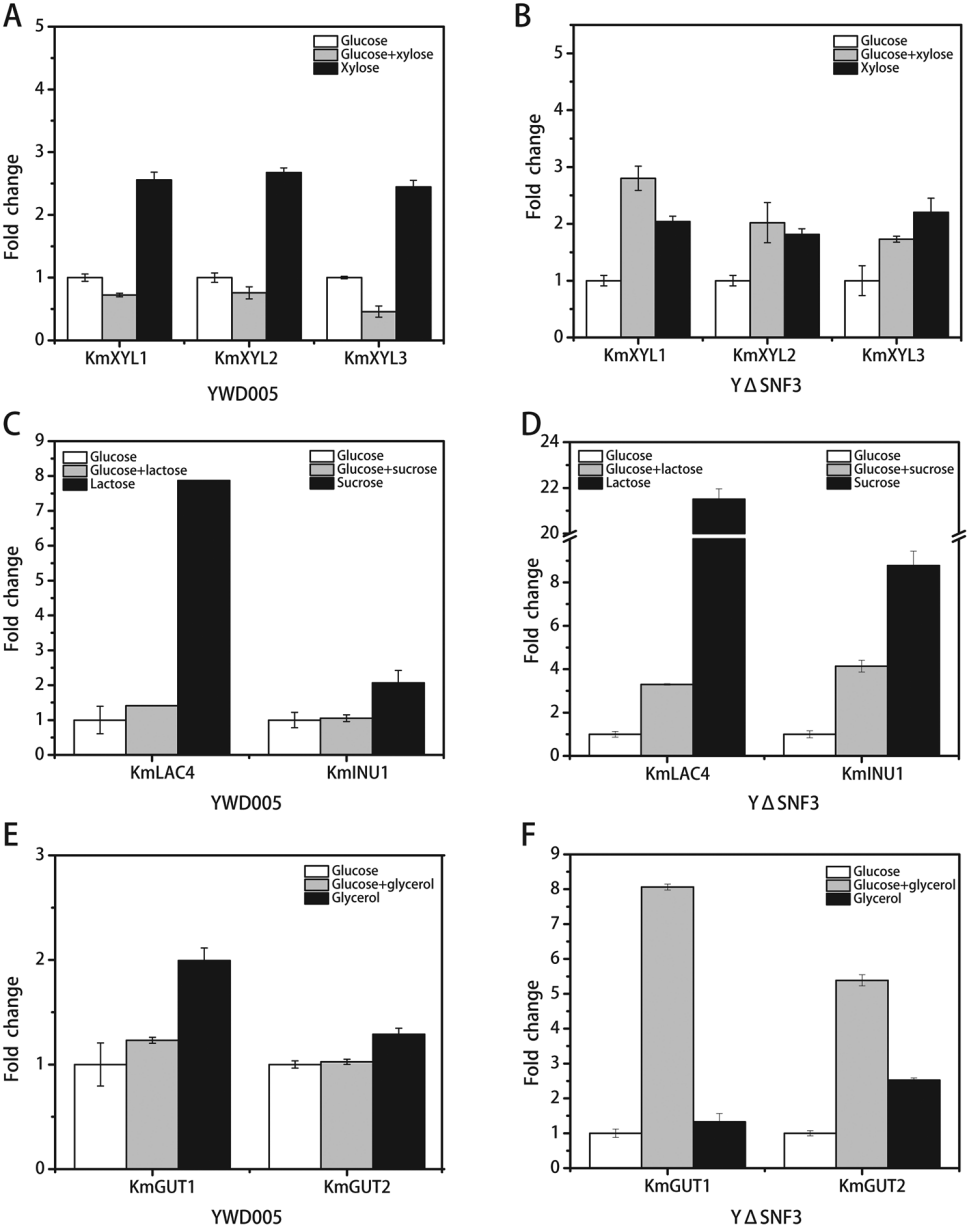
Analysis of alternative carbon sources utilization gene expression by RT-PCR. The expression levels of xylose utilization genes (**A** and **B**), lactose or sucrose utilization genes (**C** and **D**), and glycerol utilization genes (**E** and **F**) were analyzed. YWD005 served as a non-disrupted control. The error bars represent the standard deviations calculated from triplicate experiments

Firstly, the expressions of *KmXYL1*, *KmXYL2*, and *KmXYL3* were analyzed. In the control strain (YWD005), the relative expression (fold-change) of *KmXYL1* in glucose, glucose-xylose, and xylose was 1:0.72:2.55 (Fig. 3A). Likewise, the relative expressions of *KmXYL2* and *KmXYL3* on glucose, glucose-xylose, and xylose were 1:0.75:2.67 and 1:0.45:2.64, respectively (Fig. 3A). Although there was no increase with the glucose-xylose mixture, the expression of these three genes was dramatically upregulated by xylose as the sole carbon source. These results indicate that *KmXYL1, KmXYL2*, and *KmXYL3* are xylose-inducible genes and are repressed by glucose in YWD005. In YΔSNF3, however, the relative expressions of *KmXYL1, KmXYL2*, and *KmXYL3* on glucose, glucose-xylose, and xylose was 1:2.80:2.03, 1:2.02:1.81, and 1:1.72:2.20, respectively (Fig. 3B). The expressions of these three genes were dramatically upregulated on the glucose-xylose mixture, suggesting that *KmXYL1, KmXYL2*, and *KmXYL3* are xylose inducible genes and are not repressed by glucose in YΔSNF3.

Secondly, the *KmINU1* expression was analyzed in the presence of glucose, glucose-sucrose, or sucrose as the carbon source. In the control strain (YWD005), the relative expression of *KmINU1* in the above carbon sources was 1:1.06:2.06. This result indicates that *KmINU1* is an inducible gene and is repressed by glucose in YWD005 (Fig. 3C). In YΔSNF3, however, the relative expression of *KmINU1* under the same conditions was 1:4.14:8.77. Compared with that on glucose, the expression of *KmINU1* was upregulated 4-fold by sucrose, even in the presence of glucose. This result indicated that *KmINU1* is an inducible gene and glucose repression is relieved in YΔSNF3 (Fig. 3D).

Additionally, the *KmLAC4* expression was analyzed in the presence glucose, glucose-lactose, or lactose as the carbon source. In the control strain (YWD005), the relative expression of *KmLAC4* on the above carbon sources was 1:1.41:7.87. This result shows that *KmLAC41* is an inducible gene and is repressed by glucose in YWD005 (Fig. 3C). In YΔSNF3, however, the relative expression of *KmLAC4* under the same condition was 1:3.29:21.50 (Fig. 3D). The expression of *KmLAC4* increased more than 3-fold in the lactose-glucose mixture. These results indicate that *KmLAC4* is an inducible gene and glucose repression is relieved in YΔSNF3.

Finally, the expressions of *KmGUT1* and *KmGUT2* were analyzed in the presence of glucose, glucose-glycerol, or glycerol as carbon sources. In the control strain (YWD005), the relative expressions of *KmGUT1* and *KmGUT2* on the above carbon sources were 1:1.23:1.99 and 1:1.02:1.29, respectively (Fig. 3E). Interestingly, the expression of *KmGUT1* and *KmGUT2* did not increase as significantly as other genes even in the absence of glucose in the medium. In YΔSNF3, however, the relative expression of *KmGUT1* and *KmGUT2* under the same conditions were 1:8.06:1.32 and 1:5.38:2.52, respectively (Fig. 3F). Unexpectedly, the expressions of *KmGUT1* and *KmGUT2* with only glycerol were lower than those with glucose-glycerol. Although the reason was unclear, we speculated that the better growth with glucose-glycerol than with glycerol led to this difference. Furthermore, the increased expression of *KmGUT1* and *KmGUT2* with glucose-glycerol suggests that glucose repression is released in YΔSNF3.

### Overexpression of the glucose transporter genes restored the glucose utilization ability but maintained the release of glucose repression

The function of *KMAR_50343*, *KMAR_50346* and *KMAR_10531* was confirmed by expression in the HXT-null strain (*S. cerevisiae* EBY.VW4000; Gift from Professor Eckhard Boles) [26]. YEBYVW4000-50343 and YEBYVW4000-50346, which expressed *KMAR_50343* and *KMAR_50346*, respectively, grew well on a synthetic dropout (SD) solid medium with glucose as the carbon source, whereas YEBYVW4000-10531, which expressed *KMAR_10531*, did not grow (Supplementary Fig. S4). These results indicate that KMAR_50343 and 50,346 encode glucose transporters. There are more than 20 putative sugar transporter genes in the *K. marxianus* genome. Other than *KmSNF3*, which is different from other genes, several lactose transporter genes (*LAC12*) have been studied [36], and Donzella et al. characterized the pentose transporters in *K. marxianus* [35].

In the absence or lower concentration of glucose, the glucose repression of *S. cerevisiae* is released, which allows Rgt1 to bind the promoters of several *HXT* genes and represses their expression [3]. These phenomena are consistent with the transcriptome analysis results of YΔSNF3. The decreased expression of *KMAR_50343* and *50,346* which transports glucose probably led to a decreased glucose uptake which was consistent with the reduced glucose utilization ability of *K. marxianus* YΔSNF3 (Fig. 2C).

**Fig. 4:**
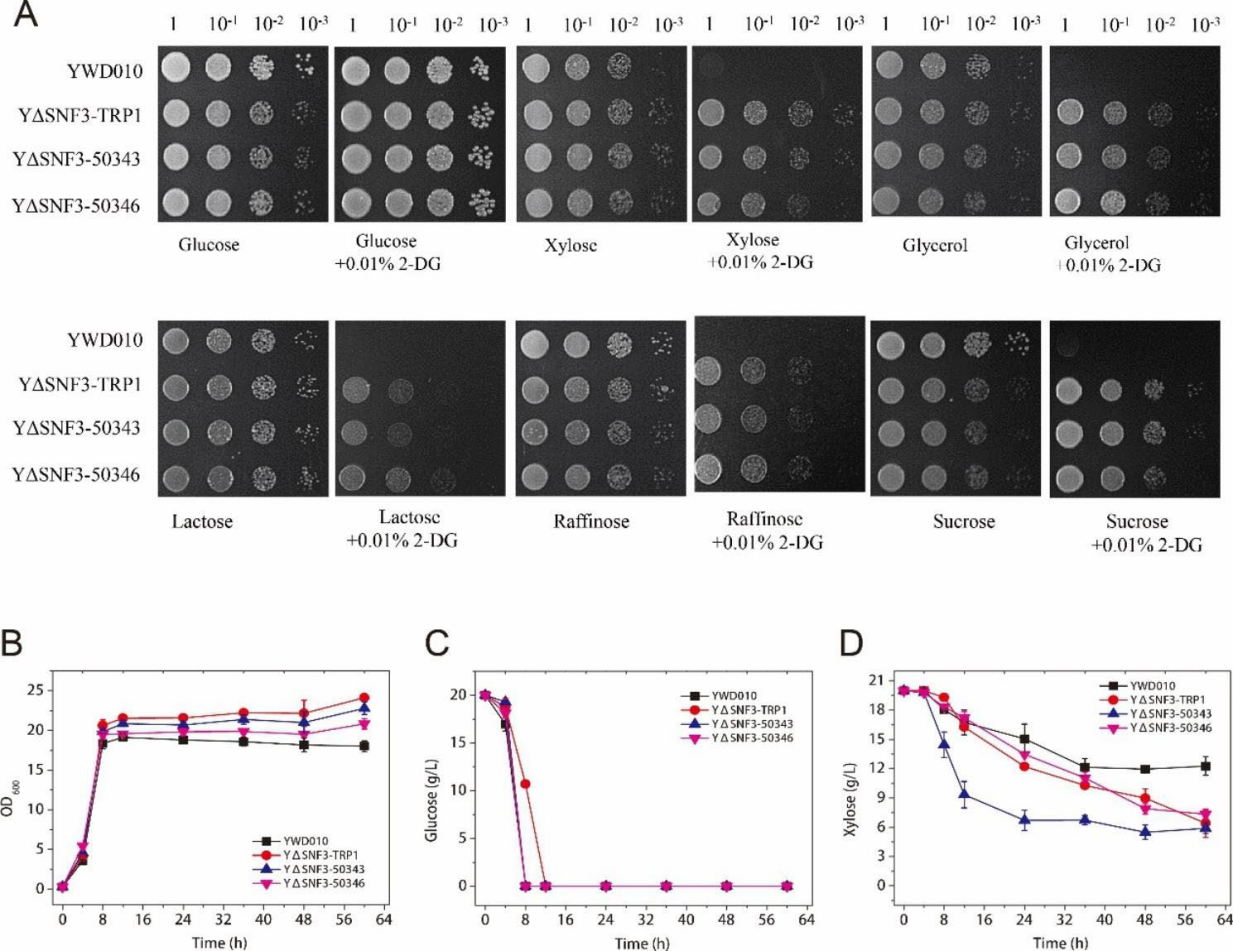
Evaluation of glucose repression in the strains overexpressing the glucose transporter genes. (**A**) Glucose repression assay, (**B**) Growth curve, (**C**) Glucose consumption, (**D**) Xylose consumption. YWD010 and YΔSNF3-TRP1 served as a non-disrupted and *SNF3*-disrupted control, respectively. The error bars represent the standard deviations calculated from triplicate experiments

Subsequently, *KMAR_50343* and *KMAR_50346* were overexpressed in YΔSNF3 to evaluate their effects on glucose uptake and glucose repression. As shown in Fig. 4A, YΔSNF3-50343 and YΔSNF3-50346 grew as well as YΔSNF3-TRP1 in alternative carbon sources and 2-DG, and better than YWD010. These results indicated that the release of glucose repression was maintained even when the glucose transporter gene in YΔSNF3 was overexpressed. Here, to maintain the same auxotrophic type, YWD010, in which *URA3* and *TRP1* were complemented, was used as a control.

The glucose utilization ability of the strains expressing glucose transporters was improved. YWD010, YΔSNF3-TRP1, YΔSNF3-50343, and YΔSNF3-50346 were inoculated into liquid YPDX medium and cultivated at 42 ^o^C to evaluate their growth and sugar utilization abilities. Compared with YWD010, the amount of biomass (final OD_600_) of YΔSNF3-50343 and YΔSNF3-50346 increased but was still lower than that of YΔSNF3-TRP1 (Fig. 4B). Interestingly, the overexpression of *KMAR-50,346* and *KMAR_50343* recovered the glucose consumption-ability of the YΔSNF3 strain (Fig. 4C). These strains consumed all glucose in 8 h, the same as YWD010, whereas YΔSNF3-TRP1 took 12 h. The xylose consumption of all *KmSNF3* disrupted strains was better than that of YWD010 (control). YΔSNF3-TRP1, YΔSNF3-50343, and YΔSNF3-50346 consumed 13.58, 14.12, and 12.67 g/L xylose, respectively, in 60 h, in contrast to YWD010, which consumed 7.73 g/L in 60 h. The xylose consumption rates of YWD010, Y△SNF3-TRP1, Y△SNF3-50343, and Y△SNF3-50346 were 0.25 g/(L·h), 0.39 g/(L·h), 0.66 g/(L·h), and 0.33 g/(L·h), respectively. Moreover, YΔSNF3-50343 consumed 10.66 g/L xylose in 12 h with a xylose consumption rate of 0.87 g/(L·h) (Fig. 4D). In short, the overexpression of the glucose transporter genes led to similar glucose consumption as the control (YWD010), whereas glucose repression release was maintained and xylose utilization was enhanced (Fig. 4D). The reason that YΔSNF3-50343 consumed xylose faster than other strains was not clear. It is possible due to the expression level and affinity of *KMAR_50343* as a sugar transporter. More xylose consumption in these strains than YΔSNF3 and YWD005 (Fig. 2) was possibly due to the difference of auxotrophy.

In *S. cerevisiae*, the repression of glucose transporter expression is also accompanied by glucose derepression, however, glucose repression is restored with the increase in glucose uptake [8]. These reports suggested that the decreased expression of glucose transporters leads to glucose repression, although the mechanism is obscure. However, via the overexpression of *KMAR_50343* (Fig. 4), the glucose consumption of YΔSNF3-50343 was similar to that of the control strain YWD010 (Fig. 4C) with glucose repression released (Fig. 4A). We also attempted to determine the intracellular glucose level; however, the concentration was low and no obvious difference was detected between YΔSNF3-50343 and YΔSNF3 (Data not shown). This was possibly due to the fast metabolism of glucose in *K. marxianus* cells. As a result, improved glucose uptake did not restore glucose repression.

### The disruption of *KmGRR1* also released glucose repression, but disruption of *KmMTH1* or *KmRGT1* did not

After the disruption of *KmSNF3* was analyzed, the disruption of downstream genes was also evaluated. The disruption of *KmGRR1* (YΔGRR1) partially released glucose repression. YΔGRR1 grew better on all of the alternative carbon source plates in the presence of 2-DG than the control strain, YWD005, but worse than YΔSNF3 (Fig. 2A). YΔGRR1 showed similar growth rate (Fig. 2B) and a similar glucose consumption ability to YWD005 in liquid medium, and all glucose was consumed in 8 h (Fig. 2C). However, when only xylose was used as the carbon source, the xylose utilization by YΔGRR1 was weak (Fig. 2D and E). YΔGRR1 accumulated more biomass than YWD005 in the presence of glucose-xylose as the carbon source (OD_600_ = 16.46 and 15.17, respectively). The xylose-utilization ability of YΔGRR1 improved when the glucose-xylose mixture was used as the carbon source. YΔGRR1 consumed 12.05 g/L of xylose in 24 h, which was faster than that by YWD005 (Fig. 2H), although its glucose consumption rate was almost the same as that of YWD005 (Fig. 2G). Due to the complicated regulation of GRR1 as a ubiquitin-protein ligase and the weak xylose utilization ability of YΔGRR1 with xylose as a sole carbon source, *KmGRR1* was not further studied in this work.

As shown in Fig. 2A, the growth of the *Kmmth1* or *Kmrgt1* strain (YΔMTH1 or YΔRGT1) on all plates was similar to YWD005. Therefore, the disruption of *KmMTH1* or *KmRGT1* did not release glucose repression. The growth of YΔMTH1 and YΔRGT1 in the presence of glucose in liquid medium was similar to that of YWD005 (Fig. 2B). The glucose consumption rates of these three strains were similar and all glucose was consumed in 8 h (Fig. 2C). The growth of YΔMTH1 and YWD005 were similar when either were in the xylose or glucose-xylose mixture (Fig. 2D-H). The final cell density of YΔMTH1 was 19.83 (OD_600_) in xylose, and 12.82 in the mixed carbon source. The growth of YΔRGT1 in xylose was slower than that of YWD005 initially and finally accumulated more biomass (OD_600_ = 21.62), whereas the growth of YΔRGT1 and YWD005 was similar in the glucose-xylose mixture (Fig. 2D-H) (OD_600_ = 13.52). These results were expectable as *KmMTH1* and *KmRGT1* are required to the release of glucose repression [3].

In *S. cerevisiae*, in the presence of glucose, Mth1 and Std1 are phosphorylated and targeted for degradation via the ubiquitin-proteasome pathway by Grr1. Therefore, the disruption of *GRR1* may result in stabilized Mth1 and Std1. In *K. marxianus*, *STD1* homolog was not found in the genome. We postulated that *Kmgrr1* disruption led to reduced degradation of KmMth1 and KmRgt1; thereafter, the signal transmitted downstream of KmMth1 and KmRgt1was no- or low-glucose in the medium. The partial release of glucose repression by YΔGRR1 was achieved (Fig. 2A). Therefore, it is predictable that the disruption of *KmMTH1* or *KmRGT1* did not lead to the release of glucose repression. Subsequently, a double disruption strain (YΔSNF3-ΔRGT1) was constructed. Its growth on the alternative carbon source plate in the presence of 2-DG was repressed and similar to the growth of YΔRGT1 and the control (YWD005) (Supplementary Fig. S5). These results further proved that KmRgt1 is necessary in the release of glucose repression.

### Improving the stability of KmMth1 led to derepressed glucose repression

Considering KmMth1 and KmRgt1 could be degraded in the presence of glucose and are necessary in the release of glucose repression, the stable mutant of KmMth1 or KmRgt1 should alleviate the glucose repression. We have not enough information to construct a stable KmRgt1, however, we previously constructed a mutant of KmMth1 (KmMth1-ΔT) which is possibly stable.

**Fig. 5:**
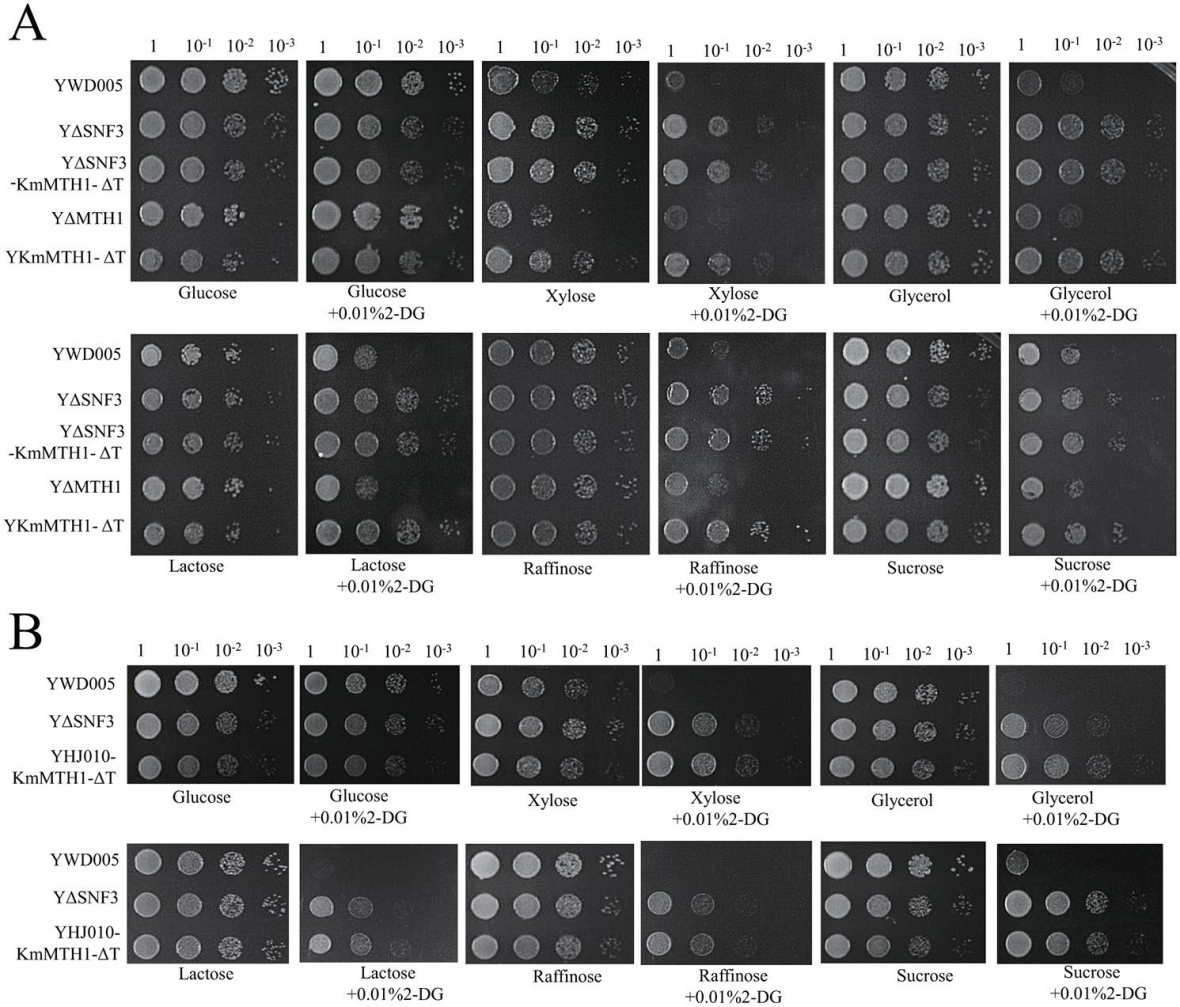
The evaluation of the KmMTH1-ΔT function in ***K. marxianus*** glucose repression. (**A**) Expression of *KmMTH-ΔT* in *Kmsnf3* or *Kmmth1* strains (YΔSNF3 and YΔMTH1); (**B**) Expression of *KmMTH1-ΔT* in *KmMTH1* wt strain YHJ010. YWD005 served as a non-disrupted control

KmMth1-ΔT was speculated to escape degradation, however, there is no detailed study on the function of this stable mutant in glucose repression. Herein, the *KmMTH1-ΔT* was overexpressed in strains YΔSNF3 and YΔMTH1. The growth of YΔSNF3-KmMTH1-ΔT in glucose or alternative carbon sources with or without 2-DG was similar to that of YΔSNF3 (Fig. 5A). These results indicated that the glucose repression of YΔSNF3-KmMTH1-ΔT was still released (Fig. 5A). After *KmMTH1-ΔT* was expressed in YΔMTH1, the growth of the resultant strain, YKmMTH1-ΔT, on various carbon sources was better than that of YΔMTH1 and YWD005 and similar to that of YΔSNF3 (Fig. 5A). That is, YKmMTH1-ΔT exhibited a glucose-repression derepressed phenotype. These results proved that a stable KmMth1 mutant transmitted the no-or-low-glucose existence signal downstream and released glucose repression.

Subsequently, the overexpression effect of *KmMTH1-ΔT* was evaluated in the wildtype strain YHJ010 in which the SRR pathway was not modified. As shown in Fig. 5B, on all alternative media, the growth of YHJ010-KmMTH1-ΔT was similar to that of YΔSNF3 and better than that of YWD005 in the presence of 2-DG. These results suggest that the stable KmMth1 mutant released glucose repression even in the wildtype strain.

### The repression of xylose utilization genes was released in YΔGRR1, YΔSNF3-50343, YΔSNF3-50346, YHJ010-KmMTH1-ΔT

**Fig. 6:**
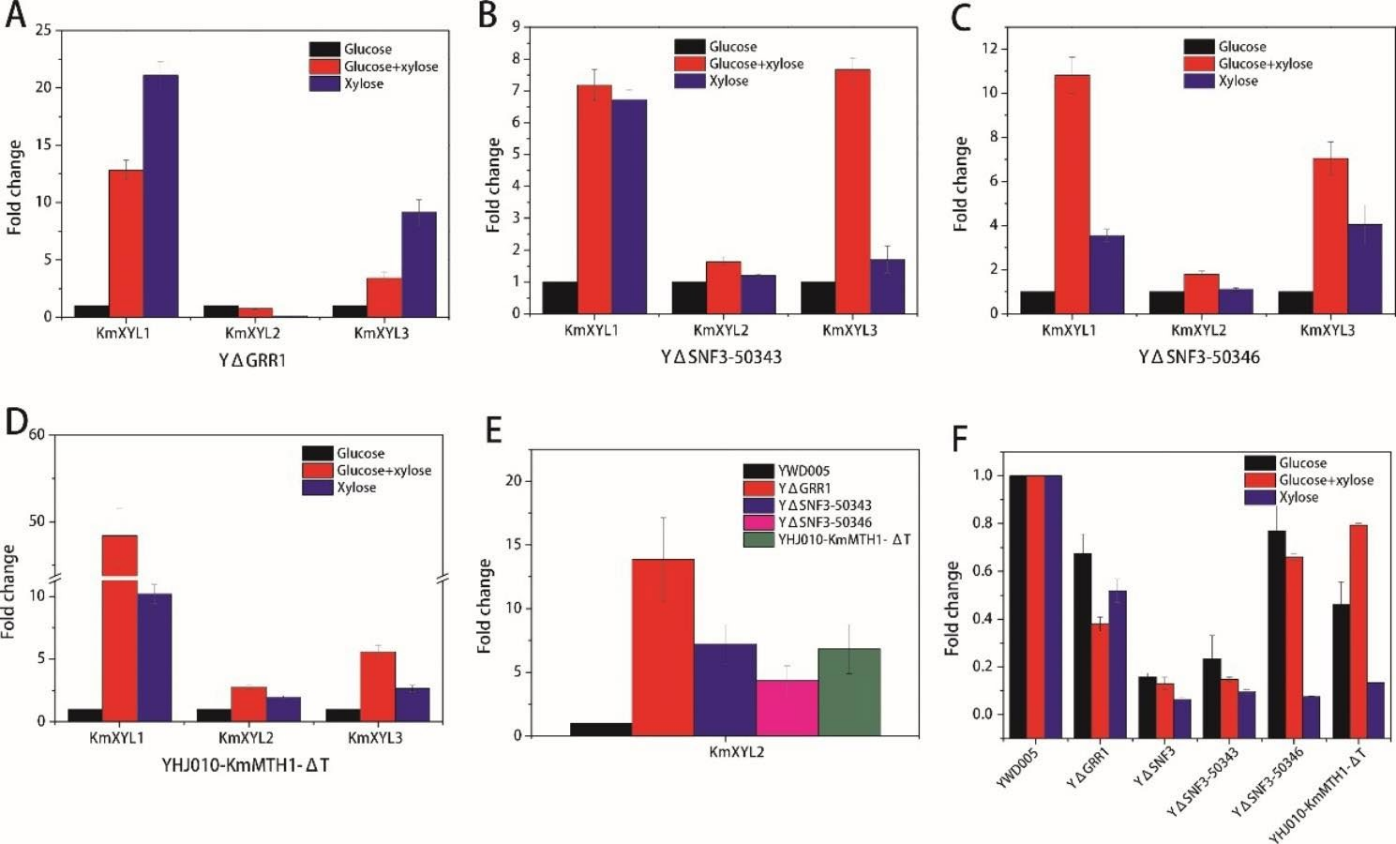
RT-PCR analysis of the expression of xylose utilization genes and ***KmHXK1*** in glucose repression-released strains. The expression of *KmXYL1*, *KmXYL2*, and *KmXYL3* in YΔGRR1 (**A**), YΔSNF3-50343 (**B**), YΔSNF3-50346 (**C**), YHJ010-KmMTH1-ΔT (**D**), expression of *KmXYL2* in above strains with glucose as carbon source (**E**), the expression of *KmHXK1* in each glucose-repression released strains (**F**). YWD005 served as a non-disrupted control. The error bars represent the standard deviations calculated from triplicate experiments

The spot assay results indicated that glucose repression in YΔGRR1, YΔSNF3-50343, YΔSNF3-50346, and YHJ010-KmMTH1-ΔT was released (Figs. 2 and 4, and 5), however, the derepression to the gene expression for alternative carbon source was not confirmed. Herein, the expressions of *KmXYL1*, *KmXYL2*, and *KmXYL3* in the presence of glucose, glucose-xylose, or xylose was determined by RT-PCR to verify their release. As shown in Fig. 6A-D, the expressions of *KmXYL1* and *KmXYL3* in all the evaluated strains were higher in the xylose or glucose-xylose mixture than in only glucose. Consistent with the spot assay results, the increased expression of these genes in the glucose-xylose mixture suggested that glucose repression was released. However, the expression of *KmXYL2* in YΔSNF3-50343, YΔSNF3-50346, and YHJ010-KmMTH1-ΔT did not increase as much as *KmXYL1* and *KmXYL3*. We compared the *KmXYL2* expression in YΔGRR1, YΔSNF3-50343, YΔSNF3-50346, and YHJ010-KmMTH1-ΔT with that in the control strain (YWD005) in the presence of glucose (Fig. 6E). The expression level of *KmXYL2* in YΔGRR1, YΔSNF3-50343, YΔSNF3-50346, and YHJ010-KmMTH1-ΔT was 13.85, 7.23, 4.34, and 6.82, respectively, of fold change, compared to those in YWD005. Therefore, the increased basal expression of *KmXYL2* in the presence of glucose led to a relatively low change in expression in the presence of xylose. Although *KmXYL1* and *KmXYL3* expression increased in the presence of xylose, the *KmXYL2* expression in YΔGRR1 did not increase even in the presence of xylose. The relative expressions of *KmXYL2* in YΔGRR1 with glucose, glucose-xylose, or xylose was a 1.00, 0.75, and 0.12 fold change, respectively (Fig. 6A). The decreased expression of *KmXYL2* in the presence of xylose explains the weak growth of YΔGRR1 with xylose as sole carbon source (Figs. 2D and 6A).

**The crosstalk of SSR pathway and Mig1-Hxk2 pathway in*K. marxianus***.

**Fig. 7:**
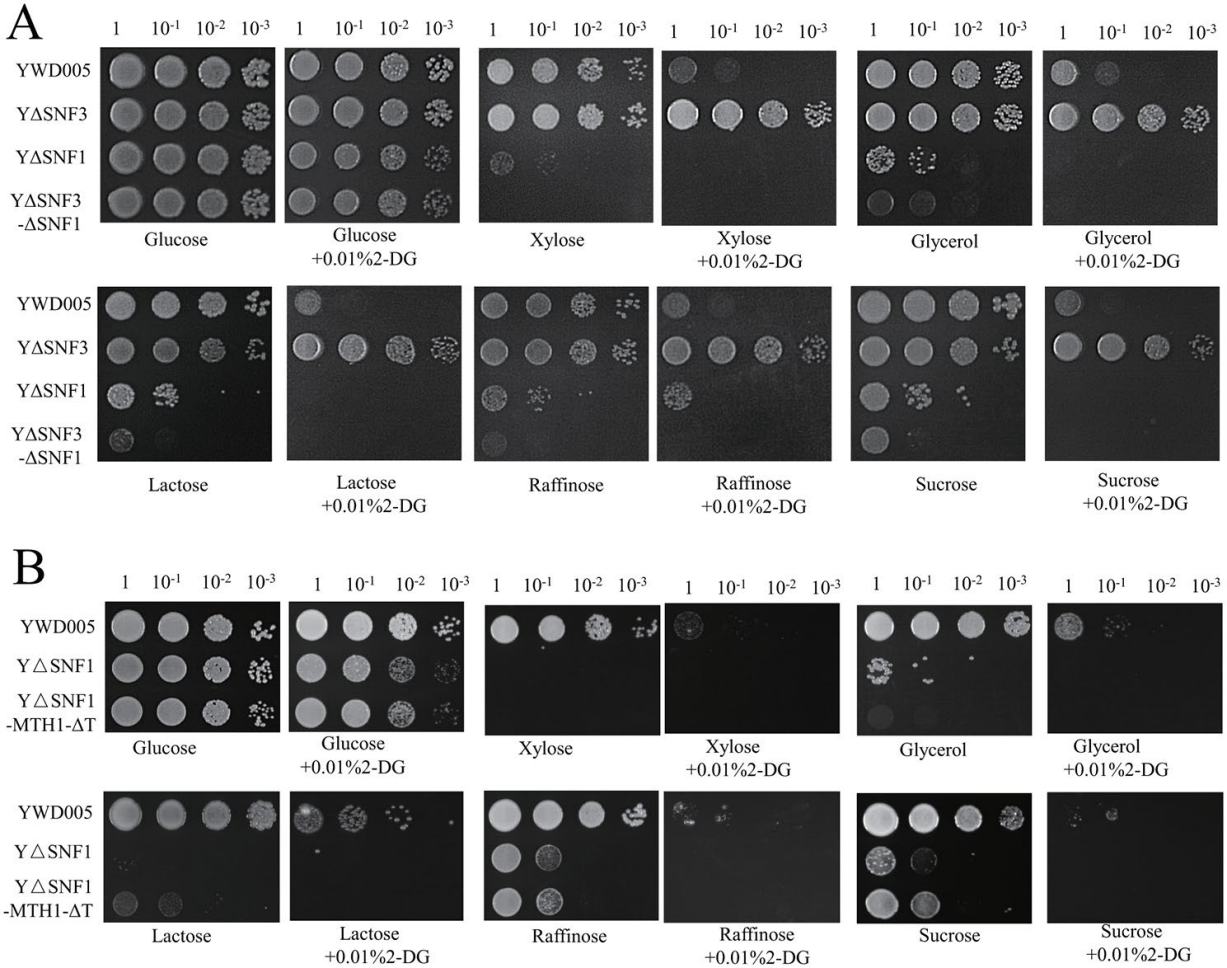
Evaluation of the crosstalk of the sensor/receptor repressor (SRR) and Mig1-Hxk2 pathways in glucose repression. The glucose repression of YΔSNF3-ΔSNF1 (**A**) and YΔSNF1-KmMTH1-ΔT (**B**) was evaluated. YWD005 served as a non-disrupted control

In our previous study, we investigated the Mig1-Hxk2 pathway and proved that the disruption of the sole hexose kinase gene (*KmHXK1*) releases glucose repression of *K. marxianus*, although the glucose utilization ability decreased markedly [14]. Therein, the disruption of *KmSNF1* (YΔSNF1) led to a defective alternative carbon source utilization ability [14], and the expression of the genes for alternative carbon sources was constitutively repressed. Here, to study the crosstalk between the two pathways, *KmSNF1* in the YΔSNF3 glucose-repression released strain was disrupted. As shown in Fig. 7A, YΔSNF3 grew better than YWD005 on the alternative carbon source plate in the presence of 2-DG, and YΔSNF1 grew weakly on the alternative carbon source plate without 2-DG. The double disruption strain (YΔSNF3-ΔSNF1) grew the worst with or without 2-DG on the alternative carbon source plate. YΔSNF3-ΔSNF1 restored the glucose repression and showed a more severe glucose repression compared to the *Kmsnf1* strain (YΔSNF1) (Fig. 7A). In *S. cerevisiae*, the cells of *SNF1* disrupted strain are hypersensitive to 2-DG due to the reduced expression of low-affinity, high-capacity glucose transporters [37]. The disruption of *KmSNF3* also led to reduced expression of those glucose transporters with rich transcript abundance (see the transcriptome section). Therefore, double disruption strain was more sensitive to 2-DG.

Furthermore, *KmMTH1-ΔT* was expressed in strain YΔSNF1 to evaluate the crosstalk between the two pathways (Fig. 5B). In Fig. 5, the overexpression of *KmMTH1-ΔT* released the glucose repression in *K. marxianus* although the genes in the glucose-sensing pathway were not modified. However, the repressed growth on the plate containing 2-DG and alternative carbon sources indicated that YΔSNF1-MTH1-ΔT expressing *KmMTH1-ΔT* in YΔSNF1 maintained glucose repression (Fig. 7B). Therefore, either via the SRR or MIG1-HXK2 pathways, *KmSNF1* was required for the release of glucose repression.

In *S. cerevisiae*, in the presence of high glucose concentrations, Snf1 is inactive, and leads to the non-phosphorylation of transcription factor Mig1 and the repression of the expression of genes involved in the utilization of alternative carbon sources [7]. When glucose is limited, Snf1 is activated and phosphorylates Mig1 and Rgt1. Phosphorylated Mig1 leaves the promoter of the alternative carbon source utilization genes and releases glucose repression. Phosphorylated Rgt1 binds to the promoters of *HXT* and *HXK2* and represses their expressions [38]. This interaction between Rgt1 and Snf1 kinase is critical for the derepression of *HXT* expression and plays an important role in overall glucose repression [7]. However, how Rgt1 regulates the glucose repression to the expression of alternative carbon utilization genes remains unclear. Herein, the disruption of *KmSNF1* restored the glucose repression that was derepressed by the disruption of *KmSNF3* or overexpression of *KmMTH1-ΔT* (Fig. 7).

### The expression of *KmHXK1* decreased in glucose repression-released strain

As mentioned above, *HXTs* and *HXK2* are repressed by *RGT1*, and Hxk2, which is a component of the Mig1-Hxk2 pathway, is verified to regulate the glucose repression in *S. cerevisiae*. In our previous study, the disruption of *KmHXK1* releases glucose repression in *K. marxianus* [14]. Thus, the expression level of *KmHXK1* in glucose repression released strains was analyzed.

Transcriptome analysis indicated that *KmHXK1* expression in YΔSNF3 was significantly downregulated (Log_2_FC: -2.20; Supplementary Table S3) when the glucose-xylose mixture was used as a carbon source, compared to that in YWD005. To evaluate whether the glucose signal obtained in the SRR pathway led to glucose repression via the interaction of KmRgt1 and KmHxk1, the expression level of *KmHXK1* in *K. marxianus* YΔSNF3, YΔGRR1, YHJ010-KmMTH1-ΔT, YΔSNF3-50346, and YΔSNF3-50343 was determined with RT-PCR. When glucose was used as the sole carbon source, the *KmHXK1* expression in YΔGRR1, YΔSNF3, YΔSNF3-50343, YΔSNF3-50346, and YHJ010-KmMTH1-ΔT was lower than that in YWD005 (Fig. 6F). When xylose was used as the sole carbon source, the expression of *KmHXK1* in glucose repression-released strains was further decreased, with the exception of YΔGRR1. However, when glucose and xylose were the carbon sources, the expression of *KmHXK1* in the glucose repression-released strains was relatively higher than that with xylose as the carbon source, although it was still lower than that in YWD005. With glucose, glucose-xylose, and xylose as carbon sources, the expression level (fold change) of *KmHXK1* in YΔSNF3 was the lowest and approximately 0.16, 0.13, and 0.06, respectively, in contrast to the 1.00 of YWD005 (Fig. 6F). These results indicated that in glucose repression-released strains, *KmHXK1* expression was repressed.

**Fig. 8:**
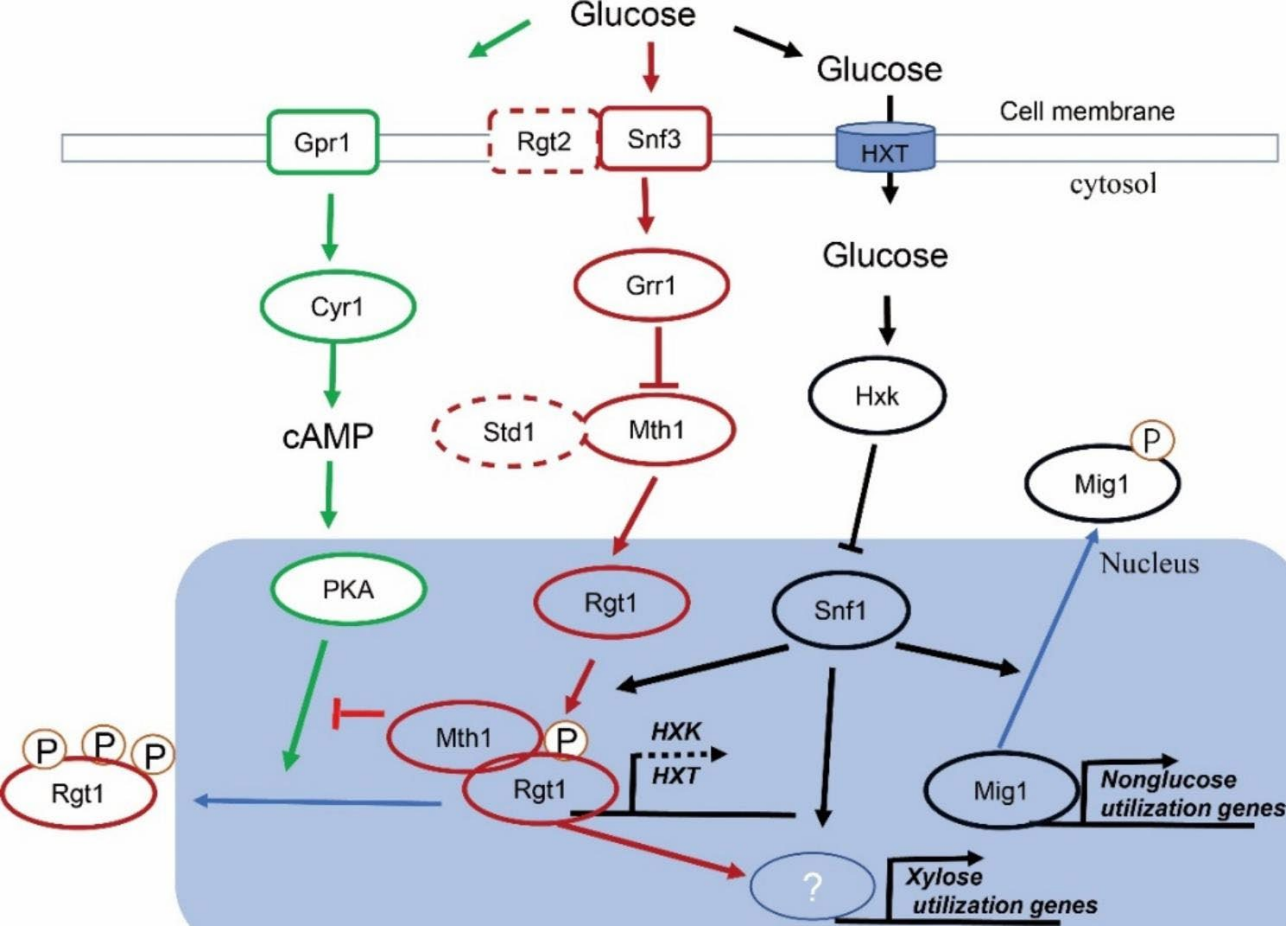
The schematic representation of proposed mechanism for glucose repression in the SRR pathway of***K. marxianus***. Red frame: SRR pathway, Green frame: cAMP/PKA pathway, Black frame: Mig1-Hxk2 pathway. Dash frame: not found in *K. marxianus*

Based on the above results, we propose a mechanism for glucose repression in the SRR pathway in *K. marxianus* which is similar to that in *S. cerevisiae* but with several new properties. KmMth1 is degraded when KmSNF3 senses glucose, mediated by KmGrr1. KmRgt1 is then hyperphosphorylated by PKA and degraded, accompanied by glucose repression activation (Fig. 8). When no or low glucose is sensed, or *KmSNF3* is disrupted, a no-glucose signal is transmitted to the downstream of the KmSnf3 pathway, leading to the obstruction of KmMth1 degradation, which in turn blocks the hyperphosphorylation of PKA. The expression of *HXT* genes is then repressed by KmRgt1 to conserve the cellular resources. Next, the *KmHXK1* expression is repressed by KmRgt1, and KmSnf1 is subsequently activated (Fig. 8). Thus, KmMig1 is deactivated and releases the glucose repression to most alternative carbon source utilizations, with the exception of xylose. As xylose utilization is not repressed by KmMig1 [14], there should be another regulator. We speculate that KmRgt1 affects glucose repression via an unknown regulator that is a repressor of xylose utilization. This unknown regulator is controlled by only KmRgt1 or by KmSnf1 and KmRgt1 together (Fig. 8), although it is possibly indirect. In this pathway, the trigger for glucose repression does not depend on the intracellular glucose concentration or glucose uptake. The decrease in glucose uptake is accompanied by glucose-repression release in the glucose repression-released strains and was more likely a result of glucose repression release (Fig. 8). The retained glucose utilization ability of *KmSNF3* disrupted strain is probably due to due to the activity of KmGlk and the retained expression of *KmHXK1*.

### *KmMTH1-ΔT* enabled the release of glucose repression to xylose utilization in *S. cerevisiae*

**Fig. 9:**
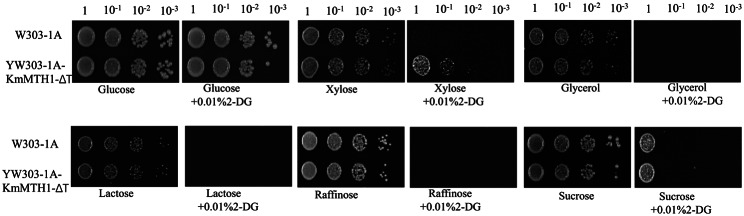
Evaluation of the effect of KmMth1-ΔT on glucose repression in *S. cerevisiae* W303-1 A

Unexpectedly, overexpression of *KmMTH1-ΔT* also released glucose repression to xylose utilization in *S. cerevisiae*. *KmMTH1-ΔT* was expressed in *S. cerevisiae* W303-1 A and YW303-1 A-KmMTH1-ΔT was obtained. As shown in Fig. 9, the growth of YW303-1 A-KmMTH1-ΔT was similar to that of W303-1 A in all the carbon sources without 2-DG, and the growth became weak on the plate of glycerol, lactose, raffinose, or sucrose with 2-DG (Fig. 9). Surprisingly, when xylose was used as the carbon source, YW303-1 A-KmMTH1-ΔT grew better than W303-1 A in the presence of 2-DG (Fig. 9), indicating that the glucose repression of xylose was released in *KmMTH1-ΔT* expression strain YW303-1 A-KmMTH1-ΔT.

Generally, the xylose utilization ability of *S. cerevisiae* is poor, although the glucose transporter system takes up xylose [39]. Furthermore, xylose reductase and xylitol dehydrogenase displayed low activity abilities in *S. cerevisiae*. However, xylose can still be converted into xylitol and xylulose [39]. Xylulose is used in most *S. cerevisiae* strains [40], and xylose utilization is enhanced by a mixed substrate and yeast extract [41]. After a longer culture time (3 days), the growth of *S. cerevisiae* in the presence of xylose was also detected (Fig. 9). Additionally, the response to the overexpression of *KmMTH1-ΔT* in the presence of xylose or other carbon sources were different. These results suggested that the glucose repression to xylose utilization may be different from that of other alternative carbon sources in *S. cerevis*iae. In *S. cerevisiae*, there are two paralogous regulatory proteins Mth1 and Std1, which binds Rgt1 to regulate the utilization of alternative carbon source. However, the function of Mth1 is not regulated as Std1 [42]. Thus, the xylose utilization is probably regulated by Mth1 indirectly in *S. cerevisiae*.

## Conclusion

Several novel properties of the SRR pathway have been identified in *K. marxianus*. First, the disruption of the sole glucose sensor *KmSNF3* gene, disruption of *KmGRR1*, or overexpression of the *KmMTH1* stable mutant in the SRR pathway of *K. marxianus* released the glucose repression on the utilization of an alternative carbon source. Second, unlike *S. cerevisiae*, the disruption of *KmSNF3* did not lead to a defect in its growth in the presence of glucose, and a better growth in the presence of xylose was achieved. Third, the glucose assimilation ability was restored via the overexpression of the glucose transporter gene, and glucose repression was not restored by enhanced glucose uptake. As such, glucose uptake and repression were not interdependent. Therefore, it was possible to construct glucose repression-released and a maintained glucose utilization ability in *K. marxianus.* Finally, the overexpression of *KmMTH1-ΔT* also released glucose repression on xylose utilization in *S. cerevisiae*, but not to other alternative carbon sources. Altogether, these results indicate that the mechanism of glucose repression to xylose utilization is novel and different from that of other carbon sources in yeasts, enabling future metabolic engineering approaches to obtain more robust strains for industrial fermentation.

## Electronic supplementary material

Below is the link to the electronic supplementary material.

Supplementary Material 1: **Fig. S1** The growth of SNF3 disrupted strain (YΔSNF3) on 0.1% or 2% glucose medium. **Fig. S2** The ethanol accumulations of YΔSNF3 with glucose (A), xylose (B) or mixture of glucose and xylose as carbon source (C). **Fig. S3** GO and KEEG enrichment analyses of the RNA-seq results. **Fig. S4** The growth of S. cerevisiae strains which expressed the transporter genes. **Fig. S5** Evaluation of the effect of KmRGT1 disruption on glucose repression. **Table S1** plasmids used in this study. **Table S2** Primers used in this study. **Table S3** RNA-seq results.

## Data Availability

All data generated or analyzed during this study are included in this published article and its supplementary information files.
