# Supplementary material for: The novel properties of Kluyveromyces marxianus glucose sensor/receptor repressor pathway and the construction of glucose repression-released strains
Source: Microb Cell Fact. 2023 Jul 10;22:123. doi: 10.1186/s12934-023-02138-7 (PMC10331984; doi:10.1186/s12934-023-02138-7)
Supplement: Supplementary file 1 — Supplementary Material 1: Fig. S1 The growth of SNF3 disrupted strain (YΔSNF3) on 0.1% or 2% glucose medium. Fig. S2 The ethanol accumulations of YΔSNF3 with glucose (A), xylose (B) or mixture of glucose and xylose as carbon source (C). Fig. S3 GO and KEEG enrichment analyses of the RNA-seq results. Fig. S4 The growth of S. cerevisiae strains which expressed the transporter genes. Fig. S5 Evaluation of the effect of KmRGT1 disruption on glucose repression. Table S1 plasmids used in this study. Table S2 Primers used in this study. Table S3 RNA-seq results. [file 12934_2023_2138_MOESM1_ESM.docx]

**Supplementary information: Supplementary Figures and Tables**

**The novel properties of *Kluyveromyces marxianus* glucose sensor/receptor repressor pathway and the construction of glucose repression-released strains**

Lingya Wang^1#^, Anran Wang^1#^, Dongmei Wang^1,3^, Jiong Hong ^1,2,3^

^1^School of Life Sciences, University of Science and Technology of China, Hefei, Anhui 230027, P. R. China

^2^ Hefei National Laboratory for Physical Science at the Microscale, Hefei, Anhui 230026, P. R. China

^3^ Biomedical Sciences and Health Laboratory of Anhui Province, University of Science & Technology of China, Hefei 230027, China.

*Corresponding:

Jiong Hong, [hjiong@ustc.edu.cn](mailto:hjiong@ustc.edu.cn), Phone: +86 551-63600705

Dongmei Wang, [dmwang09@ustc.edu.cn](mailto:dmwang09@ustc.edu.cn), Phone: +86 551-63607932

^#^ equally contributed.


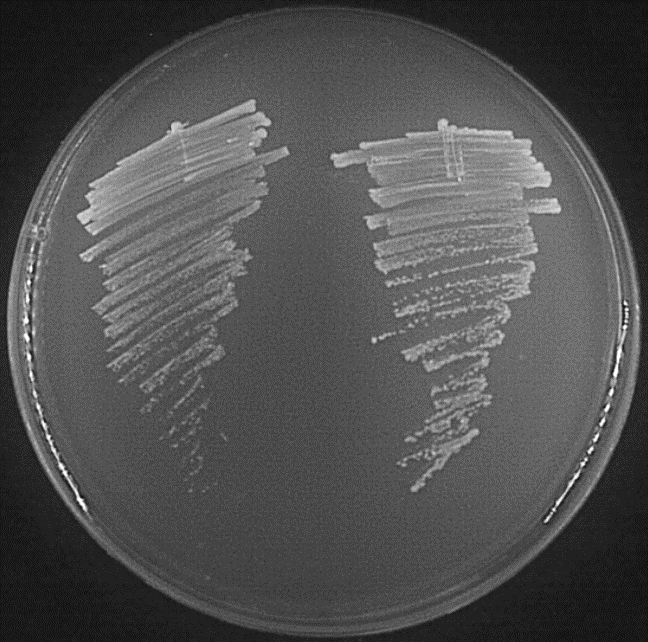


Y ΔSNF3

YWD005

0.1% glucose


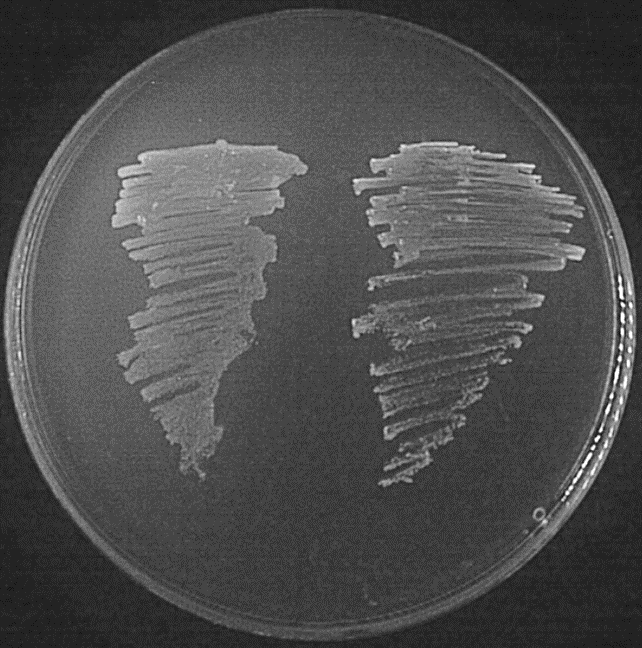


2% glucose

Y ΔSNF3

YWD005

**Fig. S1 The growth of SNF3 disrupted strain (Y ΔSNF3) on 0.1% or 2% glucose medium.** YWD005 was the control strain.


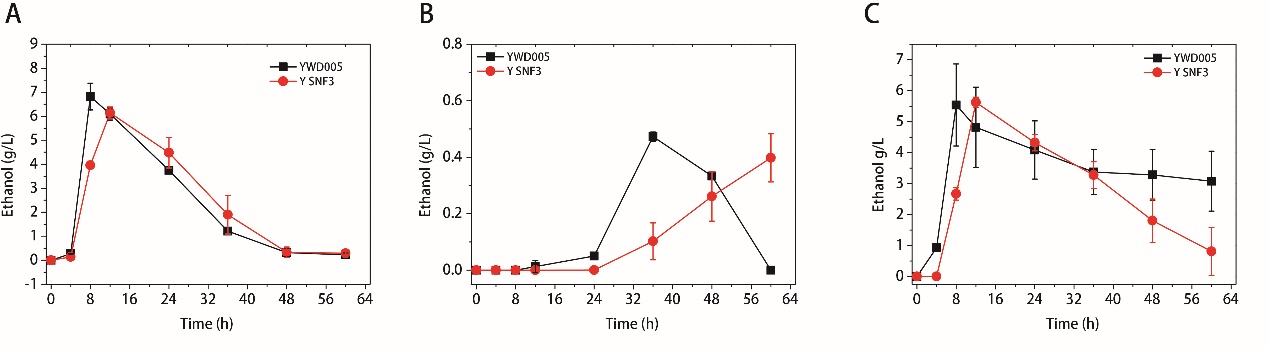


**Fig. S2 The ethanol accumulations of YΔSNF3 with glucose (A), xylose (B) or mixture of glucose and xylose as carbon source (C).**


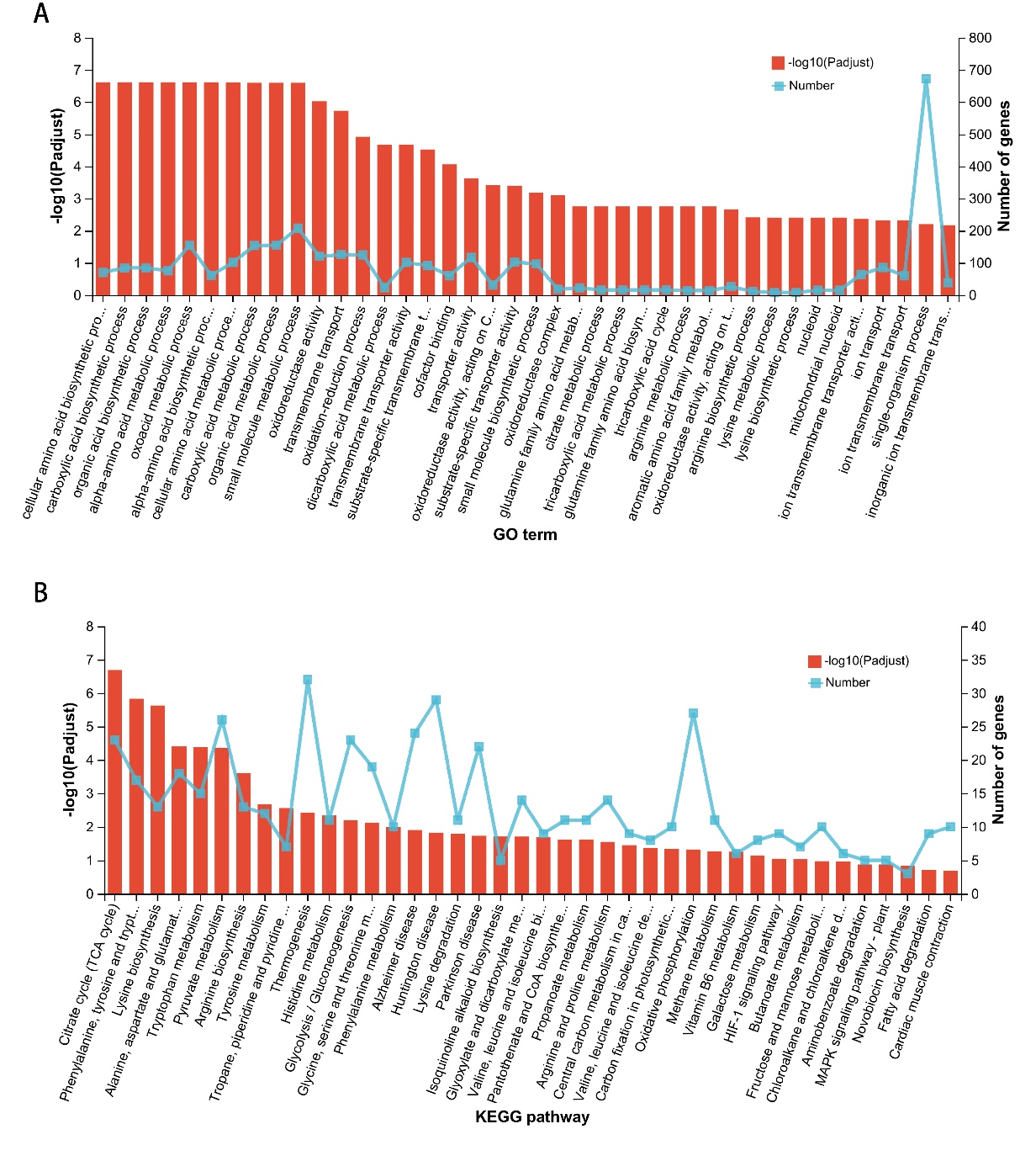


**Figure S3. GO and KEEG enrichment analyses of the RNA-seq results**. A. GO enrichment analyses and B. KEGG enrichment analyses in the comparison group of YΔSNF3 vs YWD005 in the presence of glucose and xylose. The horizontal axis represents the GO terms or KEGG pathway name, and the vertical axis represents the number of differentially expressed genes in this GO term or KEGG pathway.


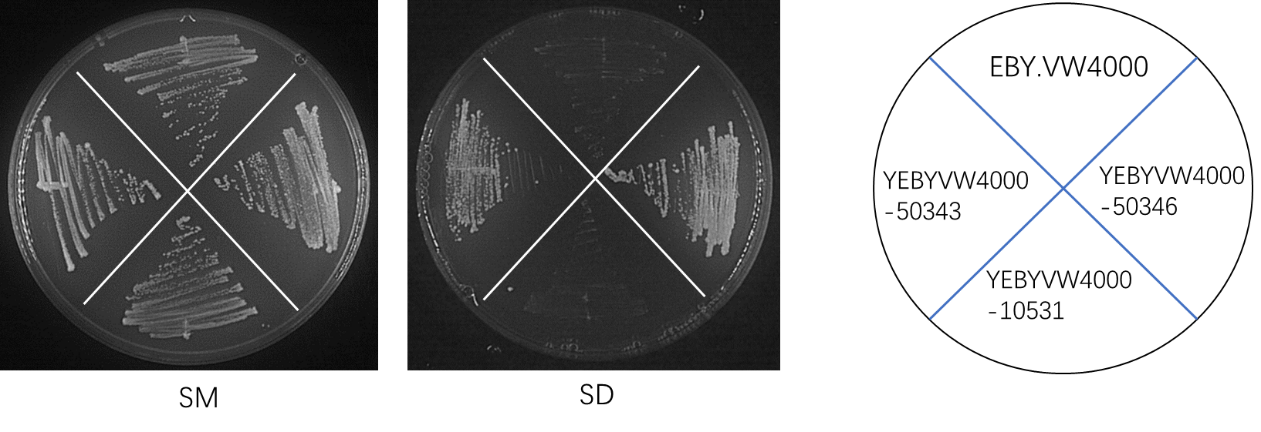


**Fig. S4 The growth of *S. cerevisiae* strains which expressed the transporter genes.** The synthetic media with maltose (SM) or glucose (SD) as the carbon source were used for evaluation.


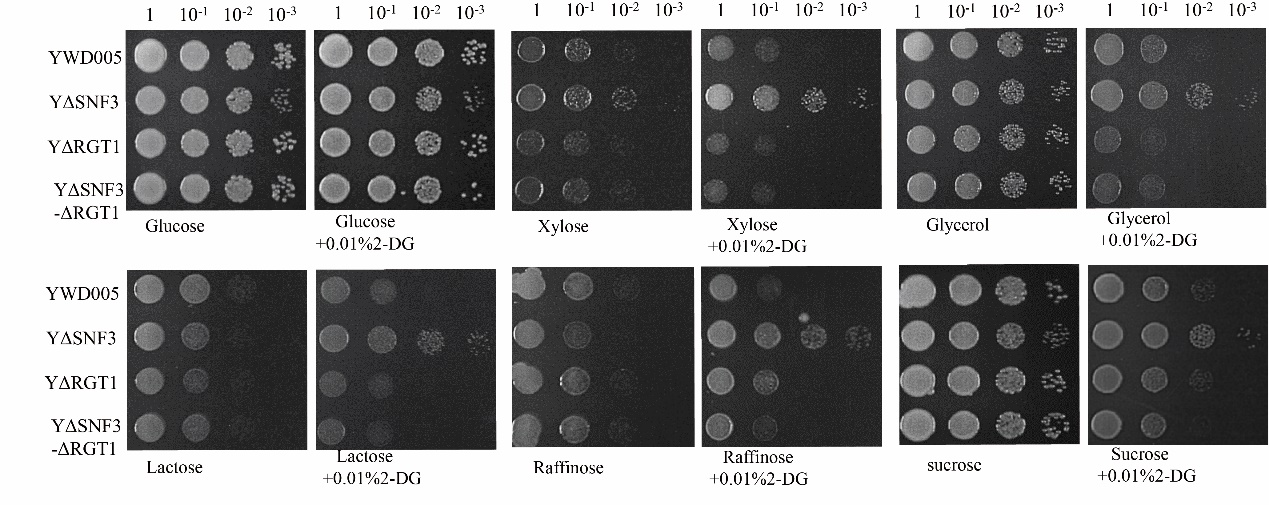


**Fig. S5 Evaluation of the effect of *KmRGT1* disruption on glucose repression.** YWD005 served as a non-disrupted control.

**Table S1 plasmids used in this study**

| Plasmid | Description | References |
| --- | --- | --- |
| YEUGAP | *ScURA3*, P*_KmTDH3_*_,_ T*_KmTDH3_* | [37] |
| YEGAP | *ScTRP1*, P*_KmTDH3_*_,_ T*_KmTDH3_* | [37] |
| pMD18T-ΔScURA3 | *ScURA3*disruption cassette | [37] |
| pKmSNF1-U | *ScURA3, KmSNF1* disruption cassette | [12] |
| pKmSNF3 | *KmSNF3* in PGEM-T easy | [12] |
| pKmSNF3-U | *ScURA3, KmSNF3* disruption cassette | [12] |
| pZB013 | *ScURA3*, P*_KmTDH3_*-*KmMTH1-ΔT*-T*_ScTDH3_* | [30] |
| pKmMTH1 | *KmMTH1* in PGEM-T easy | This study |
| pKmMTH1-U | *ScURA3, KmMTH1* disruption cassette | This study |
| pKmGRR1 | *KmGRR1* in PGEM-T easy | This study |
| pKmGRR1-U | *ScURA3, KmGRR1* disruption cassette | This study |
| pKmRGT1 | *KmRGT1* in PGEM-T easy | [12] |
| PKmRGT1-U | *ScURA3, KmRGT1* disruption cassette | [12] |
| YEGAP-10531 | *ScTRP1, KMAR_10531 expression* | This study |
| YEGAP-50343 | *ScTRP1, KMAR_50343 expression* | This study |
| YEGAP-50346 | *ScTRP1, KMAR_50343 expression* | This study |

**Table S2** P**rimers used in this study.**

| Primer | Sequence* (5’🡪3’) | |  |
| --- | --- | --- | --- |
| KmSNF3F | | aacatcgatttcgggtgtat | |
| KmSNF3R | | gtccttgcgtgtggtgta | |
| KmMTH1F | | CATTCACTTACCTATATATCCCGTTCTTGAC | |
| KmMTH1R | | GTCAATCTGTTTCTGAAGCTC | |
| KmGRR1F | | ATGGAGTCACAGCAGCCTTCTGGAGGTAAC | |
| KmGRR1R | | TTATTCATCTATGATCTGATCATCAGTTTC | |
| KmRGT1F | | ATTTTCAGACTTCTCGGAAG | |
| KmRGT1R | | CTATACTAAGTCCTGATCGGC | |
| dKmGRR1F | | GCGAATAAAGTTCCATTTCCACTGCTCG | |
| dKmGRR1R | | CTCTCTCATCTCTCATTGACGGCAGTG | |
| dKmSNF3F | | tgtgaagtagtgaattggtg | |
| dKmSNF3R | | atgttcacaactttgggg | |
| dKmMTH1F | | CGGTTGGTGGTGGTGAAACGAACATCTTC | |
| dKmMTH1R | | GCAACCGATTAAAGCAGAATAAAATACG | |
| dKmRGT1F | | GGTTGGAGTTGCTGACTGA | |
| dKmRGT1R | | CTCGTTGCAATCTTGTTCT | |
| SCURA3-SMAI-F | | TCCCCCGGGTATTTAGAAAAATAAACAAATAG | |
| SCURA3-SMAI-R | | TCCCCCGGGAATGCGTACTTATATGCGTC | |
| Km50343-ECORI-F | | GCTGAATTCATGTCTGAAGCTGCTGCTGA | |
| Km50343-NOTI-R | | GTTGCGGCCGCTTAATGCTTCATCATGGCCTTG | |
| Km50346-ECORI-F | | GCTGAATTCATGTCCAATCAATTAACGGATAC | |
| Km50346-NOTI-R | | GTTGCGGCCGCTTAGTTCTTCTTGAAGGACATGC | |
| KM10531-ECORI-F | | ATCGAATTCATGTCATTGAAAGACAAGATTTTG | |
| KM10531-NOTI-R | | GTTGCGGCCGCTTAGTTAGAGTTTGAGTTTGAG | |
| KmSNF1F | | CTGTCGGAAGAAGAATGGAG | |
| KmSNF1R | | TAATAACGCGGGGATCAGC | |
| RT-XYL1-F | | GGTATCGCCGTCATTCCAAAGTC | |
| RT-XYL1-R | | TGGTCCAAGCCGTTGATCTCC | |
| RT-XYL2-F | | GGTAAATCGCCTGAAATCGCTATTG | |
| RT-XYL2-R | | ACATCATCTCTACCCATACCCACTTG | |
| RT-XYL3-F | | CGCAAGGACGCCAAGAATATCG | |
| RT-XYL3-R | | AGGCACCACCGACGAAGAAC | |
| RT-INU1-F | | TGTAACCACCGTCCAACGAG | |
| RT-INU1-R | | CTGGTGCGTTCAGTGGTAGT | |
| RT-LAC4-F | | GCTTCGTTACCCAAGGACCA | |
| RT-LAC4-R | | AATCCAGAGTACGAGGTCGC | |
| RT-GUT1-F | | AGCCAATCGAGTTCAGCGAT | |
| RT-GUT1-R | | TGTCTCAGCGTTTCCAGTCA | |
| RT-GUT2-F | | TCAAGCGTAATGGCAGAGCA | |
| RT-GUT2-R | | AGCCAAGGCAACAGGAAGTT | |
| RT-HXK-F | | TTGAAGGACATCTACGACTGGAAGG | |
| RT-HXK-R | | GCGATGATAGCAGCACCAACAC | |
| RT-ACT-F | | CTGTCTGGATTGGTGGTTCTA | |
| RT-ACT-R | | TTCGTCGTATTCTTGCTTTGAG | |

* Restriction sites are underlined.

**Table S3 RNA-seq results**

| **Seq ID** | ***Gene*** | **Description** | **YWD005**  **tpm** | **YΔSNF3**  **tpm** | | **log_2_FC**  **(YΔSNF3/YWD005)** | |
| --- | --- | --- | --- | --- | --- | --- | --- |
| **Sugar transporter** | | | | | | |  |
| KMAR_50027 | *KmHGT1* | high-affinity glucose transporter | 6.74 | 18.92 | | 1.62 | |
| KMAR_50342 | *KmHXT2* | hexose transporter 2 | 21.78 | 15.96 | | -0.32 | |
| KMAR_50343 | *KmHXT2* | hexose transporter 2 | 1606.97 | 135.28 | | -3.48 | |
| KMAR_50344 | *KmHXT2* | hexose transporter 2 | 7.96 | 13.17 | | 0.85 | |
| KMAR_50345 | *KmHXT2* | hexose transporter 2 | 45.32 | 7.15 | | -2.53 | |
| KMAR_50346 | *KmRAG1* | low-affinity glucose transporter | 1325.97 | 2.87 | | -8.27 | |
| KMAR_50347 | *KmRAG1* | low-affinity glucose transporter | 1.93 | 27.76 | | 3.98 | |
| KMAR_30579 | *KmSTL1* | sugar transporter STL1 | 12.6 | 32.83 | | 1.51 | |
| KMAR_10527 | *KmHGT1* | high-affinity glucose transporter | 0.1 | 0.98 | | 3.48 | |
| KMAR_10528 | *KmHGT1* | high-affinity glucose transporter | 1.31 | 2.74 | | 1.19 | |
| KMAR_10529 | *KmHGT1* | high-affinity glucose transporter | 1.04 | 3.52 | | 1.89 | |
| KMAR_10530 | *KmHGT1* | high-affinity glucose transporter | 2.04 | 47.49 | | 4.66 | |
| KMAR_10531 | *KmHGT1* | high-affinity glucose transporter | 10.04 | 2729.2 | | 8.22 | |
| KMAR_20788 | *KmLAC12* | lactose permease | 6.07 | 46 | | 3.05 | |
| KMAR_30701 | *KmLAC12* | lactose permease | 3.6 | 22.04 | | 2.74 | |
| KMAR_80407 | *KmHXT9* | hexose transporter HXT9 | 8.22 | 8.94 | | 0.12 | |
| KMAR_80370 | *KmHXT14* | hexose transporter HXT14 | 0.13 | 0.46 | | 1.82 | |
| KMAR_80005 | *KmLAC12* | lactose permease | 2.98 | 4.35 | | 0.55 | |
| KMAR_60499 | *KmHGT1* | high-affinity glucose transporter SNF3 | 16.33 | 23.79 | | 0.54 | |
| KMAR_60179 | *KmSTL1* | sugar transporter STL1 | 63.05 | 108.14 | | 0.78 | |
| KMAR_30003 | *KmLAC12* | lactose permease | 0 | 0 | | - | |
|  |  |  |  |  | |  | |
| **Non-glucose carbon utilization** | | | | | | |  |
| KMAR_10659 | *KmXYL1* | NAD(P)H-dependent D-xylose reductase | 419.19 | 5019.93 | | 3.71 | |
| KMAR_70036 | *KmXYL2* | xylitol dehydrogenase | 21.48 | 346 | | 4.14 | |
| KMAR_80069 | *KmXYL3* | xylulokinase | 70.86 | 248.75 | | 1.94 | |
| KMAR_20319 | *KmGALK* | galactokinase | 34.87 | 123.36 | | 1.95 | |
| KMAR_50308 | *KmGAL40* | lactose regulatory protein LAC9 | 0.65 | 6.06 | | 3.35 | |
| KMAR_10413 | *KmGAL80* | galactose/lactose metabolism regulatory protein | 26.73 | 113.25 | | 2.21 | |
| KMAR_10500 | *KmINU1* | inulinase | 1187.59 | 1774.93 | | 0.70 | |
| KMAR_40111 | *KmADH1* | alcohol dehydrogenase 1 | 268.69 | 16780.15 | | 6.10 | |
| KMAR_40226 | *KmADH2* | alcohol dehydrogenase 2 | 12139.82 | 783.51 | | -3.82 | |
| KMAR_80296 | *KmADH3* | alcohol dehydrogenase 3 | 9.89 | 71.56 | | 2.99 | |
| KMAR_20152 | *Km*ADH4 | alcohol dehydrogenase 4 | 165.61 | 263.28 | | 0.80 | |
|  |  |  |  |  | |  | |
| **SRR pathway** | | | | | | |  |
| KMAR_60308 | *KmRGT1* | uncharacterized transcriptional regulatory protein YKL038W | 37.08 | 101.77 | | 1.58 | |
| KMAR_30221 | *KmMTH1* | Protein MTH1 | 20.42 | 6.18 | | -1.59 | |
| KMAR_70368 | *KmYCK 3* | casein kinase I homolog 3 | 4.38 | 11.75 | | 1.55 | |
| KMAR_70096 | *KmGRR1* | SCF E3 ubiquitin ligase complex F-box protein GRR1 | 2.76 | 3.29 | | 0.38 | |
| **MIG1/HXK pathway** |  |  | |  |  |  |  |
| KMAR_60404 | *KmHXK1* | Hexokinase_1 | | 2948.36 | 586.4 | -2.20 |  |
| KMAR_40614 | *KmGAL83* | SNF1 protein kinase subunit beta-3 | | 22.15 | 20.07 | -0.02 |  |
| KMAR_30555 | *KmSNF1* | Carbon catabolite-derepressing protein kinase | | 34.38 | 34.78 | 0.14 |  |
| KMAR_20217 | *KmPRKAG（SNF4)* | 5'-AMP-activated protein kinase subunit gamma | | 39.37 | 44.02 | 0.29 |  |
| KMAR_80318 | *KmSIP1* | Pre-mRNA-splicing factor BRR1 | | 1.9 | 1.33 | -0.39 |  |
| KMAR_40613 | *KmMIG* | Regulatory protein MIG1 | | 66.34 | 4.06 | -3.90 |  |
| KMAR_20239 | *KmTUP1* | General transcriptional corepressor TUP1 OS=Kluyveromyces lactis | | 52.19 | 74.23 | 0.63 |  |
| KMAR_80288 | *KmSSN6, KmCYC8* | General transcriptional corepressor CYC8 | | 2.79 | 4.96 | 0.95 |  |
|  |  |  | |  |  |  |  |
| **cAMP-PKA pathway** |  |  | |  |  |  |  |
| KMAR_30211 | *KmCYR1* | adenylate cyclase | | 0.45 | 0.77 | 0.89 |  |
| KMAR_10622 | *KmTPK2* | cAMP-dependent protein kinase type 2 | | 23.61 | 51.84 | 1.27 |  |
| KMAR_50230 | *KmTPK* | cAMP-dependent protein kinase type 3 | | 24.22 | 17.67 | -0.33 |  |
| KMAR_20503 | *KmBcy1* | cAMP-dependent protein kinase regulatory subunit | | 164.44 | 144.49 | -0.057 |  |
| KMAR_60271 | *KmGPR1* | G protein-coupled receptor | | 5.72 | 6.99 | 0.42 |  |
| KMAR_80220 | *KmRAS2* | hypothetical protein KLMA_80228 | | 69.92 | 42.31 | -0.59 |  |
| KMAR_60087 | *KmCDC25* | uncharacterized protein YGR203W | | 95.24 | 109.88 | 0.35 |  |
| KMAR_60421 | *KmGPA2* | G protein alpha subunit | | 2.14 | 8.76 | 2.17 |  |
|  |  |  | |  |  |  |  |
| **PPP** |  |  | |  |  |  |  |
| KMAR_50598 | *KmZWF* | glucose-6-phosphate 1-dehydrogenase | | 77.27 | 173.68 | 1.30 |  |
